# Facial mimicry in its social setting

**DOI:** 10.3389/fpsyg.2015.01122

**Published:** 2015-08-11

**Authors:** Beate Seibt, Andreas Mühlberger, Katja U. Likowski, Peter Weyers

**Affiliations:** ^1^Department of Psychology, University of OsloOslo, Norway; ^2^Centro de Investigação e Intervenção Social, ISCTE - Instituto Universitário de LisboaLisboa, Portugal; ^3^Department of Psychology, University of WürzburgWürzburg, Germany; ^4^Department of Psychology, University of RegensburgRegensburg, Germany

**Keywords:** mimicry, facial expression, EMG, cooperation, competition, mood

## Abstract

In interpersonal encounters, individuals often exhibit changes in their own facial expressions in response to emotional expressions of another person. Such changes are often called facial mimicry. While this tendency first appeared to be an automatic tendency of the perceiver to show the same emotional expression as the sender, evidence is now accumulating that situation, person, and relationship jointly determine whether and for which emotions such congruent facial behavior is shown. We review the evidence regarding the moderating influence of such factors on facial mimicry with a focus on understanding the meaning of facial responses to emotional expressions in a particular constellation. From this, we derive recommendations for a research agenda with a stronger focus on the most common forms of encounters, actual interactions with known others, and on assessing potential mediators of facial mimicry. We conclude that facial mimicry is modulated by many factors: attention deployment and sensitivity, detection of valence, emotional feelings, and social motivations. We posit that these are the more proximal causes of changes in facial mimicry due to changes in its social setting.

## Introduction

We humans have complex social lives, and our sociality is deeply ingrained in the makeup of our brains. Certainly, being able to cooperate, to lead and follow, to negotiate and to care for each other has given us an advantage over other species. Yet, how exactly we manage to coordinate, understand others' states and intentions and signal our own, still needs clarification. Of course, emotion psychologists have long been fascinated with emotional expressions, and social psychologists have been studying the influence of social situations on social behavior for over a century. However, the two fields of interest have only recently been brought together. This allows us for the first time to delineate how facial muscular reactions to the most important social stimulus, the human face, depend on the social context an individual is in, including one's traits and states, or one's relationship with the other person. In this paper, we explore facial reactions to facial expressions in order to better understand human nonverbal communication and its coordination.

The social situation in which facial mimicry typically occurs is that of a conversation. Whereas facial mimicry also occurs in other social situations such as between teacher and student, or actor and audience, the current review focuses on situations where only two individuals are involved who may or may not talk to each other. Such interpersonal encounters vary according to their setting, the states and traits of each of the interaction partners and their relationship. The current review aims at identifying the key findings as they pertain to these sources of influence.

## Theories of behavioral and facial mimicry

The term mimicry describes the often unconscious and unintentional imitation of behavior shown by an interaction partner, like posture, prosody, or facial expressions, the latter called facial mimicry. Specifically, facial mimicry means congruent facial muscular activations in response to an emotional facial expression. For example, individuals react with an activation of the *Zygomaticus major* (muscle which lifts the corners of the mouth, forming a smile, labeled Zygomaticus throughout this review) when they look at a happy face, or they react with an activation of the *Corrugator supercilii* (muscle which draws the eyebrows together, forming a frown, hereafter labeled Corrugator), when they look at a sad face. These reactions can be of very low intensity and are therefore usually registered electromyographically (EMG) by placing electrodes over the respective muscle regions (Fridlund and Cacioppo, 1986). Changes in muscular activity are typically reported as difference scores between the reaction score, i.e., the muscle activity while watching the expression, and baseline, e.g., the muscle activity during the second before stimulus onset.

Measured this way, the muscular changes begin within the first 500 ms after stimulus onset and are typically outside of conscious awareness (Dimberg and Thunberg, 1998). They can be observed even after subliminal exposure (Dimberg et al., 2000; Bailey and Henry, 2009), and it seems that they cannot be suppressed, even if one is instructed to do so (Dimberg et al., 2002; Korb et al., 2010). This suggests that facial mimicry occurs automatically as a reflex-like reaction. There is also evidence for biological preparedness, because already neonates show it (cf. Field et al., 1982; Meltzoff and Moore, 1989).

### Mimicry as pure motor copy

According to Chartrand and Bargh (1999), mimicry, both facial and gestural, is based on the *perception-behavior link*. According to this theory, perception is linked to behavior because of a common, or shared, representational system for perception and action (see also Prinz, 1990). As a consequence, the probability of a certain behavior increases with the mere observation of that behavior shown by another person, an effect also referred to as the *chameleon-effect* (cf. Lakin et al., 2003). This theoretical approach is discussed by Hess and Fischer (2013) as the *matched motor hypothesis*. Chartrand et al. (2005) describe mimicry as a universal process, which takes place in all people who can perceive and imitate behaviors, and which always represents the same meaning and function.

This view of mimicry is closely related to its postulated function. It is widely agreed that facial and other mimicry promotes affiliation and plays an important role in initiating and maintaining positive social relationships (Hess and Fischer, 2013). There is evidence that someone who mimics is liked more than someone who does not mimic, even when the mimicking vs. not mimicking agents are avatars (Bailenson and Yee, 2005). Thus, mimicry has been referred to as *social glue* (Lakin et al., 2003), which binds individuals together.

Lakin and Chartrand (2003) assume that they observed mimicry of foot shaking and face rubbing because “activating the desire to affiliate temporarily strengthened the perception-behavior link. Specifically, the desire to affiliate may cause people to pay more attention to what occurs in their social environments (i.e., they perceive more)” (p. 338). Applied to facial mimicry, this means that any situation that increases or reduces the desire to affiliate should also increase or reduce facial mimicry.

### Facial expressions carry meaning

Yet, in facial mimicry, as opposed to gestural mimicry, the facial expressions of the sender and the receiver carry specific intrinsic meaning. According to Hess and Fischer (2013), smiles, fearful and sad expressions are more affiliative than frowns and disgusted expressions. But even the same expression can mean different things. A mother's reciprocation of her child's sad expression carries more information than her reciprocation of the child's foot shaking. It may mean that the mother cares about the sadness of her child, understands and shares it, which implies a communal relationship. It may mean that the mother is sad about the child's sadness, which relates to her own feeling state. It may mean that the mother wants the child to stop being sad, an appeal, or confirm the veracity of the sad news that the child anticipated, a factual information. It may even encompass several of these aspects at the same time.

In order to understand the facial response given to a facial expression, it is therefore necessary to make assumptions about the perceiver's understanding of the situation and relationship, as well as about his or her state and intentions. This is necessarily somewhat speculative, and made more difficult by the fact that many facial mimicry studies do not assess such variables. In this review, we therefore combine a top-down approach of making reasonable assumptions about likely intentions, states and interpretations, with a bottom-up approach of searching for stable findings and plausible explanations of them. Both moderators and mediators can be informative with regard to the message conveyed by a facial expression.

As mentioned earlier, to structure this review, we focus on moderation of facial mimicry by aspects of the social situational context. Specifically, we first examine factors relating to the setting, then to personal characteristics of the perceiver and the sender (except for clinical psychopathological characteristics), and finally to the relationship between perceiver and sender.

## The setting

Imagine you attend a party with loud music. It is likely that in such a situation, you focus more on your interaction partner, and tune up your facial expressions and your facial mimicry, in order to make yourself understood despite the difficult verbal communication. Now imagine you attend a business meeting, everybody wears business attire and the conversation is about money. In such a situation, you might be much more restrained in your facial communication. The reason could be that you do not want to be “read” too easily, or that you are uncertain about the appropriate facial display.

### Social interaction

As different social settings require different facial signaling, social norms and scripts develop regarding the normal, correct or desirable facial behavior in a setting. However, in most facial mimicry studies, the setting is impoverished compared to the multitude of signals present in natural settings, with participants passively watching photos, computer-generated images or short video sequences on a computer screen, with electrodes attached to their face. A few studies have, however, investigated facial responses during interactions of two naive participants, as described next.

Hess and Bourgeois (2010), using EMG measurement of two participants concurrently, found that in such interactions, women smiled more than men, and both genders showed more Duchenne smiles than polite smiles. The latter lack an *Orbicularis oculi* activation (labeled Orbicularis), which raises the cheeks and forms crow's feet around the eyes (see, e.g., Messinger et al., 2001). Interestingly, even when participants talked about an anger episode, only smiles, but not frowns, were mimicked. Likewise, during naturalistic observations in shopping malls with direct response coding by an observer, about half of the smiles of experimenters were returned but hardly any frowns (Hinsz and Tomhave, 1991). A set of studies by Heerey and Crossley (2013) allows a comparison between a natural conversation in the lab (using facial coding with the Facial Action Coding System; FACS, Ekman and Friesen, 1978a) and a highly controlled setting involving computer-displayed “senders” (using EMG). In both studies, Duchenne smiles were reciprocated earlier than polite smiles, with muscle contractions even starting before the onset of an expected Duchenne smile (Study 2).

### Cognitive load

Another difference between lab settings and natural settings is that in lab studies, care is taken that participants do not hear or see anything that is not part of the experimental setup. Yet, in personal encounters, there is always additional stimulation: usually individuals are engaged in conversation, which can be more or less demanding, depending on the topic and the goal of the conversation. There is also typically distracting background noise, visual and other stimulation. Finally, a person may be distracted by additional tasks which have to be solved, or her own thoughts. Thus, the question is whether facial mimicry still occurs when individuals have reduced processing capacity due to cognitive load. If facial mimicry is diminished by cognitive load, then we can conclude that some aspect of the secondary task interferes with the processes leading to facial mimicry.

Regarding visual distraction, the task to indicate the color of the presented faces reduced facial mimicry (Cannon et al., 2009). A task to judge the genuineness of emotion expressions eliminated facial mimicry, and showed instead Corrugator activity as a function of task difficulty (Hess et al., 1998). Yet, when the task also involved a valence judgment of the expression, facial mimicry remained intact. A more recent study asking participants to judge the authenticity of smiles found that facial mimicry was still present and that it predicted ratings of authenticity (Korb et al., 2014). Regarding auditory distraction, video sequences of Ronald Reagan's emotional expressions only evoked facial mimicry when played without sound (McHugo et al., 1985).

We conclude that mimicry of Duchenne smiles plays an important role in conversations, and that anger mimicry may be uncommon in these settings. Furthermore, focussing on another aspect of the situation than valence and emotion diminishes facial mimicry, suggesting that facial mimicry depends on emotional processing. Yet, more research in naturalistic settings is needed to understand how they influence facial communication.

## The perceiver

In conversations, individuals are always both perceiver and sender. In most experiments on facial mimicry, however, only the facial expressions of the sender are varied, which allows a clear distinction between both roles. Specifically, most research on perceiver characteristics measured facial reactions to static photos of persons or to computer generated faces, facing the perceiver with direct gaze and displaying a clear emotional expression, as described in the FACS (Ekman and Friesen, 1978a). Recently, more studies use short video sequences of actors posing the development of an expression or morphs between a neutral start frame and the full expression; we refer to these stimuli as dynamic facial expressions.

Given the importance of personal characteristics in interpersonal behavior, one can expect that across situations and relationships, some individuals tend to mimic more than others, because of different personal characteristics like cultural background, gender, and personality traits or because of their current state. Accordingly, we review evidence for modulation of facial mimicry by personal characteristics and by states.

### Personal characteristics

Considering the functionality of facial mimicry for bonding, rapport and interpersonal connection, one should expect that traits positively related to affiliative motivation enhance facial mimicry.

#### Empathy

One personal characteristic important for social rapport is empathy, which can be divided into at least two sub-constructs: emotional empathy or empathic concern (“I feel what you feel”) and cognitive empathy or perspective taking (“I understand what you feel”). Evidence for this basic distinction is provided by evolutionary (de Waal, 2008) as well as neuropsychological approaches (Shamay-Tsoory et al., 2009). Individuals high in emotional empathy or empathic concern should be more likely to show facial mimicry, because they feel with the other, and because they are motivated to show their concern (for a summary and discussion see Goldman and Sripada, 2005). Conversely, facial mimicry may enable cognitive empathy, by working as a feedback mechanism about the other person's emotional state (e.g., Neal and Chartrand, 2011).

In a first study regarding emotional empathy and facial mimicry, Sonnby-Borgström (2002) recorded Zygomaticus and Corrugator reactions to happy and angry faces in participants scoring high and low on the Questionnaire Measure of Emotional Empathy (QMEE; Mehrabian and Epstein, 1972). High compared to low scoring participants showed stronger congruent Zygomaticus and Corrugator reactions even at short stimulus presentation times (averaged for the 17, 25, 30, and 40 ms). Furthermore, correlations between facial reactions and self-reported feelings were significantly higher in the high scoring participants. In a further study, Sonnby-Borgström et al. (2003) used less presentation times and a backward masking technique and found evidence of facial mimicry at 56 ms for high empathic participants only (as determined by median split). Other presentation times (17 ms; 2350 ms) did not produce significant EMG differences.

Dimberg et al. (2011) also presented happy and angry faces to the 30 highest and the 30 lowest QMEE scorers, selected from a larger sample of students. While the low empathic participants' facial reactions did not differentiate between the happy and angry faces, the high empathic participants showed larger Corrugator reactions to angry compared to happy faces and larger Zygomaticus reactions to happy compared to angry faces. In addition, the high empathy group rated both expressions as more intense than the low empathy group. Measuring emotional empathy (QMEE) after exposure to the facial expressions, Harrison et al. (2010) found a greater differentiation of Corrugator responses between angry and sad expressions on one hand and happy expressions on the other hand for high empathic individuals.

Much less is known about the influence of cognitive empathy on facial mimicry. Likowski et al. (2011a), using the Reading the Mind in the Eyes Test (Baron-Cohen et al., 2001) found that the experimental context influences cognitive empathy–with individuals in a competitive context having less–, and that high cognitive empathy predicted specifically more happiness mimicry. Hermans et al. (2009) used extreme scorers on the Autism Spectrum Quotient (AQ, Baron-Cohen et al., 2001), which assesses, as one main component, difficulties with social interactions, presumably related to deficits in cognitive empathy. They found only for low scoring women (but not for female high scorers and men) a significant difference in Corrugator reactions to smiles (congruent Corrugator relaxation) vs. frowns (congruent Corrugator activation) and, descriptively, also a stronger Zygomaticus reaction to smiles in this group. However, the small sample (only six women in that group) precludes generalizations from this study. Sims et al. (2012) (see below) found that while low AQ-scorers showed more smile mimicry for positively conditioned faces than for negatively conditioned faces, high scorers' mimicry reactions were independent of conditioning.

In sum, the available evidence shows stronger congruent facial reactions to happy and angry faces in individuals high in emotional empathy, and suggests that they perceive emotional expressions as stronger than low empathic individuals. A tentative conclusion from the available evidence on cognitive empathy is that it may boost affiliative smile mimicry toward rewarding interaction partners. However, research with more specific measures of trait cognitive empathy is needed to corroborate these results.

#### Attachment style

Attachment styles are classifications of a person's relationship with attachment figures (Bowlby, 1969). Here, of particular interest is whether attachment needs are expressed (secure and anxious attachment) or concealed (avoidant attachment). These styles may impact affiliation behavior more generally, with avoidant adults concealing their negative reactions to negative affiliation signals. To test this, Sonnby-Borgström and Jönsson (2004) had participants watch happy and angry facial expressions with different presentation times. With a presentation time of 56 ms, representing an automatic level of processing, avoidant participants did not show any Zygomaticus responses, but compared to non-avoidant participants a tendency toward stronger Corrugator responses to angry faces. With a presentation time of 2350 ms, representing a cognitively controlled level of processing, avoidant participants showed no Corrugator response, but increased Zygomaticus activity, i.e., a smiling response, to angry faces while non-avoidant participants reacted with a congruent Corrugator activation. This suggests that avoidant individuals show enhanced anger mimicry when they are not aware of the stimulus face, but they tend to conceal this mimicry, and instead display a smile under conscious exposure conditions.

#### Social anxiety

Are individuals with fear of public speaking more sensitive to signs of social disapproval, such as an angry face? Four studies, measuring fear of public speaking with the Public Report of Confidence as a Speaker questionnaire (PRCS; Paul, 1966), investigated this issue.

Dimberg and Christmanson (1991) selected participants according to extreme scores on the PRCS. While the low fear group exhibited Zygomaticus activation to happy faces and Corrugator activation to angry faces, i.e., congruent responses, the high fear group's facial responses were not affected by the quality of the stimuli. However, in the first phase of this study, participants were presented with neutral stimuli as well as faces, which might have primed the anxious population to assess the images more cognitively and less affectively. This may therefore have led participants with extreme social fear to emotionally disengage.

Dimberg (1997) exposed women above and below the PRCS median to angry and happy facial expressions. As before, low compared to high fear women showed larger Zygomaticus responses to happy faces. However in this study, high compared to low fear women showed larger Corrugator responses to angry faces. Corrugator responses in Dimberg and Thunberg's (2007) study, which used 1 s instead of 8 s exposure in the other studies on this question, differentiated better between angry and happy photos in high-fear women than in low fear women. This was mainly due to a clear Corrugator deactivation to happy photos in the high-fear group. In this study, high fear women also showed larger Zygomaticus responses to happy faces than low fear women.

Vrana and Gross (2004) selected their participants from a pool of introductory Psychology students according to their PRCS scores. Participants for the high fear group were chosen from the top 10% scorers and participants for the low fear group from the students scoring one standard deviation around the mean. Low compared to high fear participants overall showed larger Zygomaticus activation, i.e., smiled more, especially to happy and neutral expressions. Corrugator reactions showed only a descriptive trend toward stronger activation in response to angry faces in high compared to low fear participants. Interestingly, in this study both groups showed activation of both muscles compared to baseline for happy, angry, and neutral expressions, possibly indicating amusement or cognitive effort.

Thus, three studies, all with 8 s exposure and EMG assessment, show weaker Zygomaticus activation to smiles in the high fear compared to the low fear group, suggesting reduced affiliative behavior toward strangers due to high social anxiety. The one study with 1 s exposure to expressions, however, shows stronger Zygomaticus responses to smiles in the high fear group. Also, in three studies, high compared to low fear participants reacted with stronger Corrugator activation toward angry faces. A possible interpretation is that this reaction is an emotional one indicating fear elicited by social threat. However, the *Frontalis* (muscle which raises the eyebrows) as indicator of a fearful expression has not been measured in either of the studies to validate this hypothesis. A related interpretation is that socially anxious individuals are more sensitive to all emotional expressions, as shown with short exposure times; they, however, inhibit their smile to happy faces at longer exposure times due to fear of social contact (Dimberg and Thunberg, 2007).

#### Gender

While most facial mimicry studies used male and female participants, few publications report tests for gender effects. In a study specifically concerned with participants' gender as well as the gender of the facial stimuli, Dimberg and Lundquist (1990) presented angry and happy facial expressions for 8 s each. Women reacted stronger with the Zygomaticus to happy faces. No effects of the poser's sex could be detected. This finding fits results from natural settings of more smile mimicry in female than in male dyads (Hess and Bourgeois, 2010), and of less smile mimicry by men to men passing by than in any of the other three constellations (Hinsz and Tomhave, 1991). However, Vrana and Gross (2004) failed to replicate Dimberg and Lundquist and instead found no effects of participants' gender, but an effect of the poser's sex: angry women and happy men elicited more congruent reactions.

Soussignan et al. (2013) varied participants' gender and avatars' sex and gaze direction and measured specific muscle sites for anger, fear, happiness, and sadness. Analyses revealed main effects of participants' gender not influenced by eye gaze–women compared to men showed less anger mimicry (Corrugator), and more sadness mimicry (*Depressor Anguli Oris*; the muscle which pulls the lip corners downward). For fearful and happy faces, a complex interaction emerged: women mimicked fearful expressions with averted gaze (Frontalis), while men did not mimic these expressions at all. Conversely, happiness mimicry (Zygomaticus) was stronger in the direct gaze condition, but this was only true for men. Using a similar setup with less muscle sites (Corrugator and Zygomaticus) and emotions (anger and happiness), Schrammel et al. (2009) found that female participants showed stronger Zygomaticus activation to male rather than female happy faces independent of gaze, whereas the same was not true for male participants. A further interaction involving the Corrugator, genders of perceiver and sender, and facial expression was not further decomposed.

To conclude, there is no consistent pattern of gender effects on facial mimicry. Women may show more same-sex smile mimicry and may be more attuned to environmental (fear with averted gaze) and self-disclosure information (sadness). Yet given the exploratory nature of some of these analyses, a replication is important. In addition, it seems likely that social goals and motives, as well as the concrete relationship, moderate gender effects on facial mimicry. For example, whether gender is seen as a salient intergroup dimension, whether individuals are motivated to flirt or to protect their relationships, and whether they interact with known others or strangers is likely to influence gender effects.

#### Age

Do younger and older adults differ in their facial mimicry? When presenting angry facial expressions to younger (age range 18–26 years) and older (age range 65–83 years) adults, Corrugator reactions did not depend on age (Bailey et al., 2009). In a further experiment, angry and happy faces were presented subliminally and masked with neutral faces (Bailey and Henry, 2009). There were congruent Zygomaticus and Corrugator activities to happy and angry faces, respectively, with no effect of age group.

Hühnel et al. (2014) presented a larger set of dynamic facial expressions (happy, angry, sad, and disgusting), with the expressions presented by younger and older men and women, to younger (18–30 years) and older (62–85 years) women. They also measured the activity of more muscles (Corrugator. Orbicularis, Zygomaticus; *Levator labii*, the muscle which lifts and everts the upper lip, hereafter labeled Levator). While they found similar congruent facial muscular reactions to happy (Zygomaticus and Orbicularis activation), angry, and sad expressions (Corrugator activation) for both age groups, a difference emerged for disgust expressions: only the older age group showed consistent mimicry (Levator) in response to this expression. Expression recognition accuracy in the older group was worse for happy and sad expressions; thus, the different measures show dissociation for these two expressions. No sender x perceiver interactions for the facial reactions were reported by the authors.

Thus, overall the available evidence shows more similarities than differences in facial mimicry across the investigated age groups.

#### Conclusions

Self-reported emotional empathy enhances facial mimicry of angry and happy expressions. From the reviewed studies, however, it is unclear whether this latter effect is mediated by enhanced sensitivity to emotional signals, enhanced emotional responding or enhanced emotional expressivity. Also, more evidence is needed for the role of cognitive empathy in facial mimicry.

The lack of anger mimicry in avoidantly attached individuals at longer presentation times and the lack of mimicry in individuals high in social fear in a study by Dimberg and Christmanson (1991) can be the result of chronic emotion-regulation strategies. Directing one's attention away from an emotional stimulus or re-appraising it are strategies to down-regulate negative emotions, and thereby to disengage and detach (Gross, 2014). Avoidantly attached individuals seem to detach by suppressing the activation of the attachment system (Fraley and Shaver, 1997). The present findings suggest that this only happens at longer stimulus exposure. Similarly, at longer exposure times, socially anxious individuals show a negativity bias for facial stimuli, which may be the result of an avoidance orientation (Schwarz and Clore, 1996).

To understand how these effects play out in day-to-day interactions, settings with known others have to be studied as well. Furthermore, other traits influencing social behavior, such as agreeableness, extraversion or chronic power and affiliation motives should also be tested as moderators of facial mimicry. Finally, Person x Situation x Emotion expression experiments can test whether traits influence facial mimicry especially in specific trait-relevant situations with respect to specific expressions, which would strengthen the causal models from trait to facial behavior.

Regarding the demographic categories gender, age, and culture, more studies with sufficient test power are needed. The findings for gender so far fit an evolutionary perspective, according to which women are more vulnerable to environmental threats and should therefore pick up on danger cues more easily, and men are more ready to engage in ingroup and intergroup aggression, and therefore pick up more easily on direct anger expressions (Navarrete et al., 2010).

Investigating facial mimicry in different cultures and across cultures is practically important for cultural understanding and theoretically important, in that it can help distinguish culturally learned from innate propensities. Recent evidence suggests vast differences in dynamic facial expressions between East Asians and Westerners (Jack et al., 2012). Their finding that East Asian models of several emotions show specific early signs of emotional intensity with the eyes is in line with the finding that Japanese look more at the eyes regions and US Americans more at the mouth of emotional expressions (Yuki et al., 2007).

### Current state of the perceiver

In addition to relatively stable personality factors, the perceiver's psychological and physiological state also moderates facial mimicry.

#### Fearful mood state

Participants in an experiment by Moody et al. (2007; Exp. 2) watched neutral or fear-inducing film clips and afterwards neutral, angry, and fearful expressions. In the fearful condition, participants showed fearful expressions to angry and fearful faces, as was indicated by heightened Frontalis activity already in the second half of the first second after stimulus onset. These responses may be explained by a fast and vigilant information processing style, because being in a fearful state indicates that one has to watch out for danger cues in the environment (e.g., Schwarz and Clore, 1996; Luu et al., 1998).

#### Sad mood state

Likowski et al. (2011b) induced happy and sad mood states through film clips. Afterwards, participants viewed happy, angry, sad, and neutral expression, and facial muscular activity was measured over the Zygomaticus, Corrugator and Frontalis regions. EMG analyses for the second half of the first second showed stronger mimicry of happy, sad, and angry faces for the happy compared to the sad group. Furthermore, the sad group showed hardly any significant facial muscular reactions to the emotional faces, except for a Corrugator deactivation to the happy faces. On the other hand, after having watched the happy film clip, participants showed clear signs of congruent muscular reactions to happy and sad expressions, and also a tendency to mimic anger expressions.

To conclude, facial mimicry is determined by the quality of the negative mood state and not its valence: Participants in a fearful mood showed a fear response to angry faces, while participants in a sad mood did not show any significant reaction to this expression. The latter effect can be explained by self-focused attention (e.g., Wood et al., 1990; Sedikides, 1992; Green and Sedikides, 1999).

Interestingly, participants in the sad condition reacted to happy faces with a Corrugator deactivation, just as the participants who watched the happy film clip, and they rated the sad faces as more arousing compared to the participants in the happy condition. Thus, obviously these participants still paid some attention to their social environment; however, the lack of a Zygomaticus reaction to happy faces indicates that they did not have the capacity or the motivation to show signs of affiliation.

#### Manipulation of the current state by drug application

Hormones and neurotransmitters in the body and the brain are connected with mood, arousal, motive, and need states. Thus, to understand how psychological states influence facial mimicry, it seems promising to administer drugs in double-blind, placebo-controlled designs, as a means to modify the psychological state of the participant. Hermans et al. (2006) applied testosterone or placebo to healthy female participants on two separate occasions in a double-blind, placebo-controlled, mixed factorial crossover design, and 4 h after medication, Zygomaticus and Corrugator responses to dynamic happy and angry facial expressions were measured. Testosterone, compared to placebo, decreased the congruent responses to both expressions.

This result might indicate a trade-off of status and affiliation motives, with acutely rising testosterone levels evoking the status motive and suppressing the affiliation motive (see Eisenegger et al., 2011). However, it is somewhat surprising that testosterone did not increase anger mimicry, especially given that testosterone leads to increased vigilance (van Honk and Schutter, 2007) and increased heart rate to angry faces (van Honk et al., 2001). Given that anger mimicry in status competition is potentially costly, it may well be that testosterone prepares for confrontation, but that other factors determine whether the confrontation is actually sought. Manipulating the norepinephrine system had no impact on facial mimicry (Harrison et al., 2010). We suggest that oxytocin, which is assumed to play a critical role in social cognition and behavior (cf., Churchland and Winkielman, 2012; Kanat et al., 2014), enhances facial mimicry, e.g., by enhancing the recognition of facial expressions (Shahrestani et al., 2013).

#### Conclusions

The perceiver's mood modifies facial reactions to emotional faces by changing the perception and interpretation of the social environment. A fearful reaction to angry expressions in a fearful state reflects the perceiver's internal state (see Moody et al., 2007), but it also carries a relationship meaning (I submit) and an appeal (do not hurt me). The reduced mimicry after testosterone application and in sad mood arguably have different causes. It is plausible that status motives inhibit affiliation motives, whereas a sad mood may lead to a temporary inability to engage in affiliation due to self-focused attention, not to a lack of motivation. Future studies should test mediation models for these states, and also expand the range of states examined to emotional states like anger and pride (cf. Dickens and DeSteno, 2014, for pride and behavioral mimicry). Of practical importance is furthermore the question whether and how effects of these states differ from those of chronic forms, such as neuroticism or anxiety disorders, depressive disorders, and chronically elevated testosterone levels.

## The sender

Not only perceivers, but also senders have characteristics that influence perceivers' reactions to facial expressions. Their socio-demographic variables such as senders' gender and age have been discussed briefly under perceiver characteristics. Cultural background of the sender has been studied as a determinant of group membership and will be discussed there. The senders' traits and states will influence which emotional expressions they show with which frequency, clarity and intensity. Here, we focus on two expressive features which have been experimentally investigated: eye gaze and the dynamic of the expression.

### Eye gaze

An important cue to interpreting facial expressions is gaze direction. It helps us understand who an emotional expression is directed at. Is the person afraid of me, angry at me, glad to see me (Adams and Kleck, 2003, 2005)? Thus, evaluations of expression *and* gaze direction determine the relevance of the expression (cf. Graham and LaBar, 2012).

Yet, only few studies so far have investigated whether facial mimicry is moderated by gaze. Rychlowska et al. (2012). (Exp. 3) presented photographic images of smiling people with direct and with averted gaze and found stronger Zygomaticus activation for direct gaze, which was also judged as more positive. In an experiment by Schrammel et al. (2009), avatars “walked” to the middle of the computer screen, turned to the participant or sideways, displayed a dynamically developing happy or angry expression or a neutral expression, and then left again, to the other side. Zygomaticus activity was stronger while watching happy expressions compared to angry and neutral expressions only when the smiling avatars faced the participants. Corrugator activity was higher while looking at angry and neutral compared to happy faces, and this again was more pronounced in the direct gaze condition. As described already above, the results by Soussignan et al. (2013) show higher order interactions between emotional expression, gaze direction and perceiver's gender.

### Dynamic expressions

In real-life encounters, facial expressions are often ambiguous, sometimes a mix of several emotions, often very slight and always dynamic, moving from neutral or from another emotion to the current emotional or neutral display (cf. Mühlberger et al., 2011). Yet much of the research on facial mimicry used photographic images of rather idealized emotional expressions. How valid are these findings for predicting facial mimicry in an interactive setting? To start studying this question, researchers have compared responses to still photographs of prototypical emotions with responses to dynamic video sequences or morphs, starting from a neutral expression.

Rymarczyk et al. (2011) compared muscular responses to static and dynamic (neutral to emotional) happy and angry expressions of the same actors within participants. Happy dynamic expressions produced faster and stronger mimicry than static ones. Results were less clear for angry faces: Corrugator responses were small, and the only difference was a faster Corrugator activation for dynamic angry faces.

Using FACS coding of responses to dynamic and static expressions, Sato and Yoshikawa (2007) found evidence of anger and happiness mimicry only for the dynamic versions. Sato et al. (2008) found enhanced facial EMG to happy and angry dynamic expressions, compared to the static ones, on the Zygomaticus and Corrugator, respectively. Yet they did not find differential Corrugator deactivation in response to dynamic and static smiles. In another study with a similar setup, the Corrugator showed a greater deactivation—and the Zygomaticus a greater activation—to dynamic compared to static happy expressions, yet no differences for the anger expressions were observed (Weyers et al., 2006).

In sum, dynamic, self-directed expressions generate the largest response, especially to smiles. In social encounters, emotional expressions always unfold. Compared to still images, this dynamic draws attention to the change occurring, and it is also a further cue, in combination with direct gaze, that the smile is directed at the participant. For anger expressions, the evidence is less clear, with some studies finding evidence of more anger mimicry for dynamic than for still expressions, and others not. Importantly, the available studies, while suggesting that working with dynamic stimuli increases test power, do not invalidate findings from studies with static stimuli, as static and dynamic stimuli did not produce qualitatively different effects. Apart from increasing test power, dynamic stimuli can also involve the disappearance of an expression (cf. Mühlberger et al., 2011) or the change from one expression to another. Such dynamics are frequent in interactions, yet little is known about the conditions for their mimicry.

## The relationship

The relationship between the interaction partners can be described in many ways. One of the fundamental distinctions concerns whether there is a pre-existing relationship or whether strangers interact. Pre-existing relationships can be characterized according to their predominant relational model (Fiske, 2004) or relational orientation (Clark et al., 1998) whereas for strangers, important dimensions are warmth and competence (Fiske and Fiske, 2007).

### Familiarity

Despite the obvious importance of interactions in existing relationships, we know of only two empirical publications measuring facial mimicry with long-standing relationship partners. In one study, a friend or family member vs. stranger observed the sender's disgust and pride responses to the tasks she performed (Fischer et al., 2012). A FACS analysis of the videotaped expressions revealed no disgust mimicry, and smile mimicry (here, as part of the pride display) only among intimates (friend or family). In the other, photos of the romantic partners of participants were displayed on the computer alongside photos of strangers, and EMG measures to angry expressions were taken (Häfner and Ijzerman, 2011, Study 1). Results showed increased Zygomaticus responses to the anger expression of romantic partners toward whom participants had a communal orientation. This can be interpreted as a soothing smile to regulate the partner's anger, and shows the importance of relationship variables to understand facial mimicry in existing relations. In sum, among intimates, smiles in response to smiles and to other emotional expressions seem to regulate the relationship. Mimicking negative emotions may be uncommon among intimates and in social settings. How is facial mimicry between strangers influenced by their relationship, in particular their attitudes, goals, and group membership? Having a positive attitude toward another person means assessing them as warm, friendly, good-natured and sincere. The social-cognitive content model (e.g., Fiske and Fiske, 2007) maintains that this warmth dimension of social judgments essentially answers the question: friend or foe? A person judged as warm is judged to have good intentions and goals at least compatible with one's own. According to the model, this is the case for ingroup members and close allies. Thus, attitudes, goal compatibility and group membership are naturally confounded dimensions of relationships. Nevertheless, outgroups can be seen as positive or neutral, as is typically the case between men and women, or between adults and children, and having temporarily incompatible goals in a chess game does not preclude a generally friendly relationship. It is therefore informative to manipulate these factors separately to understand how they influence facial mimicry. Next, we will review evidence regarding attitudes without a salient group membership.

### Attitudes

#### Explicit attitude manipulations

Considerable evidence supports the view that positive attitudes automatically elicit approach behavior toward objects and people, while negative attitudes automatically induce avoidance behavior (e.g., Chen and Bargh, 1999; Neumann and Strack, 2000; Neumann et al., 2004; Seibt et al., 2008). If mimicry is a means to affiliate, and thus related to approach behavior, then a positive attitude toward a person should lead to an approach orientation and hence—enhanced—mimicry, while a negative attitude should lead to an avoidance orientation and hence reduced mimicry. To test these assumptions, we manipulated attitudes experimentally (Likowski et al., 2008) and expected positive attitudes toward a person to cause stronger mimicry of facial expressions posed by that person, compared to neutral and negative attitudes; for negative attitudes, we expected no congruent or even incongruent reactions.

First, computer generated female and male neutral faces were introduced to female participants as avatars with different characters designed for computer games. Next, three character descriptions, by using adjectives, were paired to avatars with three different hair colors, and participants were asked to memorize the avatar characters with their specific traits for a later recall-task. The positive characters were described as kind, nice, likeable, and self-confident, the neutral characters as reserved, serious, calm, and neat, and the negative characters as malicious, aggressive, egoistic, and deceitful. After a recall task, showing that participants had processed the information, they watched the different characters again, however, now with happy, neutral, and sad expressions, and Zygomaticus and Corrugator activities were recorded.

Zygomaticus activation was stronger to happy faces of positive characters than to those of neutral and negative characters, with no difference in the reactions to the latter two. Additional testing for changes against baseline showed significant congruent Zygomaticus activation in response to happy faces of positive and neutral, but not of negative characters. Furthermore, while participants reacted with a congruent Corrugator activation to sad faces of positive characters, they reacted with an incongruent Corrugator deactivation to sad faces of negative characters.

Thus, facial mimicry reactions were altered after only one pairing of the avatars with the characterizing adjectives. From a functional perspective, the results make perfect sense. While a positive attitude toward a person leads to approach and affiliative tendencies and thus mimicry of happy and sad expressions, there is hardly any reason to approach and thus mimic somebody toward whom one holds a negative attitude, unless one follows a certain goal. The incongruent Corrugator deactivation to sad faces of negative characters may indicate schadenfreude, but future studies should provide more direct evidence for this interpretation. Based on the results for the negative characters, it is worthwhile to examine whether facial muscular reactions to emotional expressions can be used as a reliable and valid implicit measure in attitude research (cf., Vanman et al., 2004, for reactions to neutral expressions) and whether they can be changed by disconfirming information (cf. Gregg et al., 2006).

#### Implicit attitude manipulations

Attitudes are evaluations, and can be acquired through conditioning. Is it therefore possible to replicate the results of an explicit attitude manipulation with a conditioning procedure? To find out, Sims et al. (2012) conditioned four different neutral faces with different amounts of reward: participants won in 90 or 60% of the trials or they lost in 90 or 60% of the trials in the presence of the respective face Afterwards Zygomaticus and Corrugator reactions to faces of the same persons, now, however, with dynamic happy and angry expressions were measured. Indeed, Zygomaticus reactions to the happy faces were a positive linear function of the reward value conditioned to the respective neutral faces; in contrast, reward value had no significant effect on the Corrugator response to these expressions. For angry expressions, Zygomaticus response was unexpectedly highest in the highest loss condition, with no differences between the other three conditions. If this unexpected finding replicates, it suggests that extremely negative valence also activates the Zygomaticus, either directly or indirectly by activating muscles in its vicinity (see Sims et al., 2012).

Interestingly, even a pairing of a person's neutral face with an emotional expression of that same person changes the reactions to that person's neutral face. Aguado et al. (2013) presented neutral static expressions and immediately thereafter a happy or angry static expression shown by the same persons. They found that participants who reacted with a differential pattern, i.e., higher Corrugator activation to angry than happy faces and higher Zygomaticus activation to happy than angry faces, showed this differential pattern already to the neutral faces of the respective persons in the second half of the experiment. Thus, the affective valence of a person changes according to his or her typical facial expression toward the perceiver. In sum, attitudes formed through associative conditioning moderate facial mimicry.

### Interdependence

In order to reach our goals, we often depend on other people. We usually cooperate in work teams, compete with other individuals, teams or companies, we depend on others' fairness, and we work in hierarchies, with some individuals having more status and power than us and others less. How do these factors influence facial mimicry?

#### Cooperation and competition

First, we examine three experiments manipulating cooperation and competition with female participants. In one experiment (Likowski et al., 2011a), participants were told that they would later play a game of dice with an avatar. In the game, both players would throw dice alternately. Then, participants either read that both players would win if the sum of their final scores exceeded a certain value (cooperation), that the one with the highest score would win (competition), or that they had to reach a certain score for winning, independent of the avatar's score (neutral condition). Furthermore, participants would only see their own results; instead of the avatar's results they would see the avatar's facial expression in response to her result. Thus, the avatars' facial expressions now had a specific meaning in the situation. Next, participants played an example round.

For the EMG measurement, participants just watched happy, neutral, sad, and angry expressions, which were described as potential reactions of the avatars to their result. Finally, all stimuli were presented again with the instruction to recall the game situation and to indicate the amount of joy, sadness, and anger evoked by the faces. Then, we measured cognitive empathy (Decety and Jackson, 2004) with the “Reading the Mind in the Eyes Test” (Baron-Cohen et al., 2001; German adaption: Bölte, 2005). To evaluate the goals participants pursued, they were asked to remember the game situation and rate the importance of several aspects like *to appear likable,to have a harmonious and smooth interaction, or to understand the other person's feelings and thoughts*.

In two further studies, we subliminally primed participants with interdependence-related terms. Specifically, four primes were presented 20 times each parafoveally for 90 ms and immediately masked. Participants' task was to indicate where the “flash” had appeared. Cooperation primes were *cooperate, partner, together*, and *confederate*; competition primes were *compete* (*win* in Weyers et al., 2009), *rival, opponent*, and *competition*; and neutral primes were *neutral, background, street*, and *blackboard* (Weyers et al., 2009; Seibt et al., 2013 without cooperation condition). The EMG procedure was the same in all three studies, but no angry expressions were shown in Weyers et al. (2009). In the end, manipulation checks confirmed successful manipulations and awareness checks confirmed subliminality of the primes.

The results of these studies are summarized in Table 1. In discussing them, we will focus on results that replicated across studies. Because only Seibt et al. (2013), but not Likowski et al. (2011a), found differential interaction effects on the Orbicularis, we will not describe these results here.

**Table 1:** **Overview of effects of interdependence manipulation on facial responses to emotional facial expressions**.

	**Interdependence**					
**Study**	**Cooperation**		**Neutral**		**Competition**	
	**Zyg**	**Corr**	**Zyg**	**Corr**	**Zyg**	**Corr**
**HAPPY EXPRESSION**						
Weyers et al., 2009	–	–	Act	Rel	0	0
Seibt et al., 2013	Act	Rel	Act	Rel	0	Rel
Likowski et al., 2011a	Act	Rel	Act	(Rel)	0	Rel
**SAD EXPRESSION**						
Weyers et al., 2009	–	–	0	Act	0	0
Seibt et al., 2013	0	Act	0	Act	0	Rel
Likowski et al., 2011a	Act	Act	0	Act	0	Rel
**ANGRY EXPRESSION**						
Weyers et al., 2009	–	–	–	–	–	–
Seibt et al., 2013	Act	0	0	0	0	Rel
Likowski et al., 2011a	0	0	0	0	0	Rel

“*Act” means activation of the muscle relative to baseline, “Rel” means relaxation or de-activation relative to baseline, and “0” means no significant change relative to baseline. Parentheses indicate that the test against 0 is not significant but the difference from the other conditions is not significant either.*

For neutral priming, we found congruent Zygomaticus and Corrugator reactions to happy and sad faces, respectively; thus participants reacted to affiliative facial expressions in a congruent way (cf. Bourgeois and Hess, 2008). By contrast, participants in the neutral conditions did not show a congruent Corrugator contraction to angry faces.

We did not find enhanced congruent facial reactions to the affiliative expressions in the cooperation compared to the neutral conditions. The results for the game (Seibt et al., 2013) showed that indeed, participants primed with competition behaved more competitively than those primed with cooperation or neutral words, but the latter two groups did not differ from each other. According to van Vugt et al. (2007) women are more cooperative than men, so our female participants presumably had a cooperative stance in the control condition as well. A study including male participants could shed light on this hypothesis.

After the explicit cooperation manipulation, we observed an incongruent Zygomaticus activation to sad expressions. This activation could be fully explained by the goal to have a smooth and harmonious interaction, i.e., it can be interpreted as encouragement. Furthermore, subliminal priming for cooperation led to Zygomaticus and Orbicularis activity increases toward angry faces. These effects can be seen as evidence for a soothing smile toward a cooperation partner in order to prevent the cooperation from failing. Thus, both Zygomaticus reactions can be due to context-specific motivations.

The results of all three studies show a complete lack of Zygomaticus activation to happy faces in the competition conditions. One reason for not mimicking a happy face is a rejection of the opponent's affiliative offer (see Hess and Fischer, 2014). Another one is that a competitor's happiness signals goal progress, which has negative implications for oneself. Yet, we also found Corrugator relaxation to happy expressions in competitive contexts, which is a sign of a positive valence. Mediational analyses revealed that the lack of a congruent Zygomaticus reaction to happy faces in the competitive game condition could be explained by a decrease in state cognitive empathy: The lower the current cognitive empathy, the lower the congruent Zygomaticus response to happy faces. This suggests reduced interest in others due to reduced affiliation motivation.

Our findings for the competition conditions replicated findings by Lanzetta and Englis (1989) that competition leads to incongruent facial reactions: Specifically, participants with a competitive mind-set showed Corrugator relaxation rather than contraction to sad and angry faces. Corrugator reactions are inversely and linearly related to valence (Lang et al., 1993; Larsen et al., 2003). Thus, the competition groups presumably evaluated the sad and angry faces positively because a competitor's sad or angry face indicates an advantage for oneself. It is rather unlikely that the participants considered the anger to be a sign of aggression directed toward them, because in that case a relaxation of the Corrugator muscle, i.e., a positive affective reaction, would not make sense. Support for this interpretation comes from the significant mediations of these Corrugator relaxations to sad and angry expressions by joy in the competitive game condition.

In sum, the results show a modulation of facial mimicry by cooperation and competition. However, with one experiment (Likowski et al., 2011a) we created a specific situation, namely a game of luck in which one does not have control over the outcome. Thus, further studies should investigate a broader range of situations, for example situations in which participants have to make strategic decisions. Furthermore, it would be interesting to vary the amount of rewards that participants are promised for successfully cooperating or competing. This might modulate particularly affective reactions to the emotional expressions.

#### Fairness

A game of dice as used by Likowski et al. (2011a) is fair because the a-priori likelihood of winning is equal for both sides. Now imagine a situation in which you have to give a certain amount, and your interaction partner is free to split the amount she disposes off evenly or to keep everything. If the latter happens several times you will certainly disagree with your partner's behavior and think of it as unfair.

Hofman et al. (2012) examined the effect of such a fairness manipulation on facial mimicry. Participants were first shown morphs of developing happy and angry facial expressions. In the second part, participants received 1600 points for the upcoming game and were told that the remaining points would be exchanged for money after the experiment. They were then instructed to offer in each of the following trials 25 points to one of two neutrally looking partners who would then have 50 points in total to distribute freely. The “confederates,” however, were pre-programmed to either behave fairly (in 75% of the trials the sum was split evenly), or unfairly (in 75% of the trials the opponent kept everything). After the feedback of the trial's result, the chosen partner showed either a happy or an angry expression, identical to those used at the beginning. In the last part, all morphs from the introductory part were presented again.

During the first part of the experiment, participants showed congruent reactions to the happy and angry expressions, i.e., Zygomaticus activation to happy and Corrugator activation to angry expressions. During the second part, facial mimicry in response to happy expressions of unfair partners was reduced in comparison to the responses in the first part. And in the third part, participants showed increased anger mimicry to formerly unfair proposers and decreased anger mimicry to formerly fair proposers, while happy mimicry did not differ from the first part. Thus, the facial reactions which were mimicking responses at the beginning, *changed* according to the participants' learning experience, based on the partners' behavior during the game. The authors assume that the reduced happy mimicry after unfair offers, i.e., after a violation of social norms, is a sign of resentment, thereby not providing reinforcement of the preceding behavior.

#### Power relationship

Imagine an interaction between two individuals of different status or social power. What will happen, for example, in case the person high in power looks angrily at the person low in power? Based on power theories as well as studies concerned with emotional perception and responding, Carr et al. (2014) assumed that one's power in a relationship should shape one's facial reactions to the other's emotional expressions. Participants were primed neutrally or with high or low power by a writing task and afterwards watched the faces of target persons whose status was manipulated in power by ascribing a high or low power profession to them. The targets showed happy and angry expressions, and Zygomaticus as well Corrugator reactions were recorded. Low power participants showed Zygomaticus activation (i.e., a smiling response) to all expressions, independent of the targets' power. Their Corrugator reactions to angry and happy faces differentiated stronger for high compared to low power targets. High power participants showed Zygomaticus activation to happy faces of low, but not high power targets. And they reacted with stronger Zygomaticus activation to angry faces of high power persons. These results show that the power (status) relationship modulates facial mimicry. However, its interpretation is not always clear. For instance, why do low power individuals activate the Corrugator to angry faces of high power individuals? Future studies should measure additional muscles, like the Frontalis for fear, and disambiguate the meaning of perceived and emitted facial expressions.

### Group membership

An important part of who we are concerns our group memberships. Members of closely knit groups imitate each other and converge on group norms for clothing, hair style, accent, and non-verbal behavior. It is therefore reasonable to assume that group members show more facial mimicry among each other than toward outgroup members. Furthermore, when group identity is salient (Brewer and Gardner, 1996), group members also tend to feel group emotions following group-based appraisals (Mackie et al., 2000; Smith et al., 2007). This can be a further reason for picking up each other's emotional expressions. Finally, because group membership is important for us, being excluded from groups should motivate us to show affiliative facial behavior to get included again.

#### Ingroup vs. outgroup

Hess (2001) reported that negative racial attitudes toward members of an ethnic out-group covaried with the facial reactions to pictures of facial expressions of these out-group members: French Canadians did not mimic the happy and sad facial expressions displayed by Japanese actors, and the more negative their racial attitudes toward the members of the other ethnic group were, the more they showed incongruent facial reactions to these expressions. Specifically, they smiled at the Japanese actors' sad facial expressions and frowned at their happy ones. Participants in another study watched video sequences of emotional displays of two politicians (without sound) and negative attitudes toward the better known politician (Ronald Reagan) predicted less congruent facial reactions toward his happy expressions (McHugo et al., 1991). Yet, in a prior study, political attitudes did not modulate facial mimicry to Ronald Reagan's videotaped facial expressions (McHugo et al., 1985).

Bourgeois and Hess (2008) investigated facial reactions toward happy and angry displays by two politicians, and toward happy, sad and angry displays by alleged basketball players or non-players from an ethnic ingroup or outgroup of the participants. Happy displays were mimicked in all conditions, yet sad displays were only mimicked for faces presented as basketball-players by basketball-players (and as non-players by non-players) and angry displays were only mimicked for a politician by supporters of this politician. The context of a political debate provided a meaning of the display as directed toward the political enemy, not toward the self. Because anger mimicry in more ambiguous contexts can escalate a conflict, it is not surprising that it is avoided in such contexts. Given the lack of a smiling response to happy displays in competitive contexts (see above), it is surprising that the smiles of the competing politician were mimicked in this study. Sadness mimicry, conversely, may become more selective the more social a situation gets, because in social settings, mimicking sadness can become costly by inviting emotional sharing.

A study on French and Chinese participants' (living in France) estimates of the duration of stimulus display of angry and neutral ethnic ingroup and outgroup members sought to find evidence for differential mimicry with an indirect method (Mondillon et al., 2007). In particular, the prediction was that French participants would overestimate the duration of angry ingroup members' displays because they would tend to imitate these displays. This should lead to higher arousal, which in turn would be the proximal cause for the bias. Results confirmed these predictions. Chinese participants, however, did not show a differential estimation. For them, French and Chinese expressions may have been equally relevant, because they lived in France, leading to equal imitation of both groups. The task in this study was a non-social one, which might explain that anger mimicry presumably occurred.

van der Schalk et al. (2011) showed female psychology students angry, happy, and fearful displays of male models, allegedly also studying psychology (ingroup) or studying economics (outgroup). In a second study, they showed Dutch participants of both genders dynamic facial expressions of Dutch and other nationals of unspecified gender. Replicating Bourgeois and Hess (2008), no effect of the group manipulations was found for the mimicry of happiness displays. Conversely, participants showed more facial mimicry in response to ingroup anger and fear than to the corresponding outgroup displays, as measured by EMG in Study 1 and FACS in Study 2. The finding for sadness fits with the Bourgeois and Hess findings, yet they found no anger mimicry for the basketball ingroup. A difference between these two studies is that Bourgeois and Hess used male models (photos) and participants while van der Schalk et al. used male models and female participants in Study 1 and dynamic expressions in Study 2.

Studying teenagers' and adults' reactions to same-age and different-age video-morphings, Ardizzi et al. (2014) found enhanced ingroup mimicry for teenagers, but not for adults. Specifically, the study found enhanced Corrugator reactions in teenagers to teenagers than to adults, while adults' reactions did not differentiate between target groups. From the graphs, it becomes apparent that this difference is carried by teenargers' stronger Corrugator responses to teenagers' vs. adults' sad, fearful, and angry expressions. Both age groups, however, showed similar congruent Zygomaticus reactions to happy faces, independent of the sender's age. Contrary to these results, Hühnel et al. (2014, see above) did not observe an ingroup vs outgroup interaction effect.

#### Social exclusion

Social exclusion is a powerful social stressor leading to a wide range of cognitive and behavioral changes intended to regulate one's social relationship because of a fundamental motivation to belong with others or groups (Baumeister and Leary, 1995). Thus, one should expect that affiliative motivation increases after social exclusion, thereby promoting facial mimicry at least to affiliative expressions, and this has indeed been shown by Kawamoto et al. (2014). They used a ball-tossing game (Cyberball) to manipulate social exclusion and found stronger facial mimicry to happy faces after social exclusion compared to social inclusion, as indicated by larger Zygomaticus responses.

#### Conclusions

The described experiments indicate that group membership is a powerful moderator of the facial reactions to emotional faces. Being a member of a specific group leads to affiliative signs, i.e., smile mimicry, and also to mimicry of sad expressions of members of one's own group, the latter indicating empathy and possibly support. Regarding age groups, only teenagers, but not adults, showed ingroup effects in facial mimicry. These effects could be either due to attitudes, or to shared and non-shared group identity (cf., Schubert and Häfner, 2003). Finally, being excluded from a group increases smile mimicry, possibly indicating increased affiliative tendencies.

## General conclusions

This review examines what is known about facial mimicry in social encounters. We found that many factors that are important in social encounters moderate facial mimicry. We also discovered that this moderation is not just a matter of more or less mimicry, but that the intensity and type of facial response shown to facial displays also depend on the facial expression and the gaze direction. This result fits the observation that facial expressions carry intrinsic meaning, such that imitating a smile does not mean the same as imitating an anger expression. This makes it difficult to discover general rules for when individuals mimic what. Investigating any possible combination of setting, perceiver, relationship, interaction dynamic, expression, muscle site, and gaze direction is impractical, possibly akin to trying to predict the exact verbal reply to a particular statement somebody makes.

On the other hand, this review did show the value of isolating important modulating factors. By trying to understand how these factors influence facial mimicry, we can hope to get closer to the proximal causes and functions of facial responses to facial expressions, and thereby to a predictive model. Different approaches have been used to study the processes underlying facial mimicry. One approach is to test if emotional reactions to the stimuli covary with facial responses or mediate them. Using this approach, Likowski et al. (2011a) discovered that a positive facial response to a negative expression of a competitor was mediated by joy. That same paper reports mediation of other responses by situational goals, and of others by cognitive empathy.

Not only self-reports, but also other responses such as event-related potentials can help understand facial mimicry. Achaibou et al. (2008) found that facial mimicry covaried with early event related potentials in the EEG indicative of perceptual processing, which suggests that perception and attention modulate facial mimicry. Regarding not primarily the causes, but rather the functions of facial mimicry, several researchers have blocked facial mimicry to show that it can help in expression recognition (e.g., Oberman et al., 2007; Stel and van Knippenberg, 2008; Maringer et al., 2011; Rychlowska et al., 2014).

While these studies suggest the involvement of perceptual, attentional, emotional, and motivational processes in shaping facial responses to facial expressions, only one paper tested potential mediators of facial responses to facial displays (Likowski et al., 2011a) so the interpretation has to remain speculative. Given that many of the studies reviewed included angry and happy expressions of the sender, we will next discuss likely processes behind facial responses to these expressions.

### Responses to happy expressions

Happy expressions typically evoke Zygomaticus and Orbicularis contractions and Corrugator relaxations. These responses are rather robust to moderating influences. Even in a sad mood, with no other facial responses, Likowski et al. (2011b) observed a Corrugator deactivation to happy expressions, and this was also the only congruent facial reaction shown to competitors (Likowski et al., 2011a; Seibt et al., 2013). We suggest that this robustness is due to several processes jointly determining happiness mimicry.

First, genuine smiles act as social rewards (Heerey and Crossley, 2013), and therefore evoke a positive response and a tendency to return the reward (Sims et al., 2012). Second, smiles clearly signal a desire to get along and thereby form a solid basis for friendly relationships (Hess and Fischer, 2013; Rychlowska et al., 2015). Likewise, smile mimicry signals to the mimicker that the sender is being authentic (Korb et al., 2014), thus reinforcing affiliative motivation. Third, Corrugator relaxation and Zygomaticus contraction are not specific to happiness and can indicate any positive emotion or affect (Lang et al., 1993; Larsen et al., 2003), as well as other types of smiles, such as dominance or affiliation smiles (Niedenthal et al., 2010). And fourth, returning a smile is usually not a costly signal—no promise is made by returning a smile (with a few exceptions), such that a strong habit can develop to automatically return a smile in most circumstances.

Thus, the next interesting questions to address are not so much whether smiles are responded to with any of these muscles, but more how we can recognize which meaning the smile has (see also Niedenthal et al., 2010), how smiling behavior differs between strangers and friends, between and within groups, and which muscle indicates which aspect of the response.

### Responses to angry expressions

More puzzling are the responses to angry expressions. If the reason we show mimicry is for the social goal to affiliate with others, mimicking their angry expression does not make a lot of sense. Anger carries the meaning: “You are responsible for my negative outcome” and thereby does not exactly invite affiliation and bonding. Rather, it has been characterized as an aggressive expression (Krieglmeyer and Deutsch, 2013), which may be strategically employed to enforce norm compliance (Hofman et al., 2012). Why, then, did so many studies find anger mimicry? We suggest several explanations.

First, what looks like anger mimicry need not actually be an anger expression at all. Various studies test Corrugator to angry vs. happy expressions, thus effects can also be carried by the Corrugator deactivation to smiles. Furthermore, a contracted Corrugator can also be a sign of global negative affect (Larsen et al., 2003), disapproval (Cannon et al., 2011), incoherence (Topolinski et al., 2009), surprise (Topolinski and Strack, 2015), doubt (Sanna et al., 2002), or mental effort (Stepper and Strack, 1993; Hess et al., 1998; Strack and Neumann, 2000; Koriat and Nussinson, 2009). This goes back to (Darwin, 1955 [1872]) who characterized the frown as a reaction to an obstacle (p. 220). Thus, anger expressions can be “frowned upon” because they are surprising, impolite, and unmotivated.

Second, the less social a situation, the more individuals may allow themselves to engage in mimicry as a way to understand an expression. That is, anger mimicry may be much less common in real encounters than in lab situations (Hess and Bourgeois, 2010). Thus, it may well be that the more “serious” the anger expression of the sender is, and the more real the response, the less likely the anger mimicry. For example, communal partners smiled to angry expressions of their romantic partners, but not of strangers (Häfner and Ijzerman, 2011), and high power individuals did not show pure anger mimicry to anger expressions of other high power individuals, because they also showed Zygomaticus activation (Carr et al., 2014). This latter finding resonates with research finding a preference for complementarity in dominant and submissive postures, rather than imitation (Tiedens and Fragale, 2003). Third, anger mimicry can make sense when the anger is felt as a group emotion toward a common opponent (van der Schalk et al., 2011). And fourth, anger mimicry may also serve to deter aggression, which may explain that men are more likely to show it (Soussignan et al., 2013).

Given these various possibilities, it is important to measure these potential processes in order to understand the meaning of a particular finding.

### Methodology

Facial mimicry research always involves facial stimuli with varying expressions and a measure of facial responses. Variations in these aspects across studies complicate the comparison of results. Table 2 shows methodological differences among the studies reviewed.

**Table 2:** **Overview of studies referenced**.

**Moderation^a^**	**Study**	**Measurement^b^**	**Time per stimulus^c^**	**Measurement period^d^**	**Stimuli^e^**	**Expressions^f^**	**Source^g^**	**Sites^h^**	**N^h^**
Social inter-action; gender	Hess and Bourgeois, 2010 (Study 1)	EMG	30–625 s (*M* = 183 s)	15 s time bins	Live interaction	ha, an	n/a	Zyg, Corr, Orb, Lev	96 same sex dyads (48 f)
Social inter-action; gender	Hess and Bourgeois, 2010 (Study 2)	EMG	30–856 s (*M* = 185 s)	15 s time bins	Live interaction	ha, an	n/a	Zyg, Corr, Orb, (Lev)	72 mixed sex dyads
Social inter-action; gender	Hinsz and Tomhave, 1991	Direct coding	n/a	Relative frequencies	Live interaction	ha, an, ne	Own	KJ79	1095 (483 m, 612 f)
Social interaction	Heerey and Crossley, 2013 (Study 1)	FACES	300 s	300 s	Live interaction	Genuine and polite smiles	n/a	KS07	48 same sex dyads (24 f)
Social interaction	Heerey and Crossley, 2013 (Study 2)	EMG	4 s	2 s/2 s	Static	Genuine and polite smiles	Own	Zyg, Corr, Orb	35 (85% f, 15% m)
Cognitive load	Cannon et al. (2009)	EMG	1 s	300–1000 ms	Static	ha, an	MBFSS	Zyg, Corr	46 f
Cognitive load	Hess et al., 1998 (Study 1)	EMG	10 s	10 s	Static	ha, an, dis	JACFEE	Zyg, Corr	48 f
Cognitive load	Hess et al., 1998 (Study 2a+2b)	EMG	10 s	10 s	Static	ha, an, dis	JACFEE	Zyg, Corr, Orb, (Lev)	90 f
Empathy	Sonnby-Borgström, 2002	EMG	17 ms–6 s	Not specified	Static	ha, an	EF1	Zyg, Corr	43 (21 m, 22 f)
Empathy	Sonnby-Borgström et al., 2003	EMG	17, 56, 2350 ms	2.5 s	Static	ha, an	EF1	Zyg, Corr	61 (33 m, 28 f)
Empathy	Dimberg et al., 2011	EMG	5 s	5 s	Static	ha, an	EF2	Zyg, Corr	144 (72 m, 72 f)
Empathy; current state	Hermans et al., 2006	EMG	6 s	6 s; 500 ms time bins	Morph	ha, an, ne	EF2/KDEF	Zyg, Corr	20 f
Attachment style	Sonnby-Borgström and Jönsson, 2004	EMG	17–2350 ms	2.5 s	Static	ha, an	EF1	Zyg, Corr	62 (33 m, 28 f)
Social anxiety	Dimberg and Christmanson, 1991	EMG	8 s	8 s	Static	ha, an, ne	EF2	Zyg, Corr	30 (gender unspecified)
Social anxiety	Dimberg, 1997	EMG	8 s	8 s	Static	ha, an	EF2	Zyg, Corr	16 f
Social anxiety	Dimberg and Thunberg, 2007	EMG	1 s	1 s; 500 ms time bins	Static	ha, an	EF2	Zyg, Corr	56 f
Social anxiety; gender	Vrana and Gross, 2004	EMG	8 s	8 s	Static	ha, an, ne	EF2	Zyg, Corr	19
Gender	Dimberg and Lundquist, 1990	EMG	8 s	8 s	Static	ha, an	EF2	Zyg, Corr	48 (24 m, 24 f)
Gender; eye gaze	Soussignan et al., 2013	EMG	2 s	2 s; 100 ms time bins	Morph	ha, an, fe, sad, ne	Poser	Zyg, Corr, Front, Dep	31 (17 f, 14 m)
Age	Bailey et al., 2009	EMG	5 s	200–500 ms; 500–800 ms	Static	ha, an, ne	EF2	Corr	35 younger, 35 older
Age	Bailey and Henry, 2009	EMG	14/42 ms (young/older adults)	200–800 ms	Static	ha, an, ne	EF1	Zyg, Corr	46 younger, 40 older
Age	Hühnel et al., 2014	EMG	20 s	20 s; 1 s time bins	Video	ha, an, sad, dis	Own	Zyg, Corr, Orb, Lev	39 older f, 39 younger f
Age; ingroup vs. outgroup	Ardizzi et al., 2014	EMG	4+1 s	5 s; 500 ms time bins	Dynamic + static	ha, an, fe, sad, ne	Own	Zyg, Corr	20 teenagers; 20 adults
Current mood state	Moody et al., 2007 (Study 1)	EMG	1 s	1 s in 100 ms epochs	Static	an, fe, ne	EF2	Corr, Front	48 (6 m, 42 f)
Current mood state	Moody et al., 2007 (Study 2)	EMG	1 s	500–1000 ms	Static	an, fe, ne	EF2	Corr, Front	39 (12 m, 27 f)
Current mood state	Likowski et al., 2011b	EMG	6 s	6 s	Static	ha, an, sad, ne	Poser	Zyg, Corr, Front	60 f
Eye gaze	Rychlowska et al., 2012, Study 3	EMG	8 s	500–1500 ms	Static	ha, ne	Own	Zyg	27 f
Current state	Harrison et al., 2010	EMG	≥1 s	500–1000 ms	Static	ha, sad, an	KDEF	Zyg, Corr	40 (26 m, 14 f)
Eye gaze; gender	Schrammel et al., 2009	EMG	3 s morph, 5.5 s total	500–1000 ms after apex	Morph	ha, an, ne	Poser	Zyg, Corr	44 (22 m, 22 f)
Dynamic expressions	Rymarczyk et al., 2011	EMG	1.5 s	1,5 s; 500 ms time bins	Static/morph	ha, an	MSFDE	Zyg, Corr	27 (12 m, 15 f)
Dynamic expressions	Sato and Yoshikawa, 2007	FACS	1520 ms	2.5 s	Static/morph	ha, an	Own	AU4, 12	18 (9 m, 9 f)
Dynamic expressions	Sato et al., 2008	EMG	1520 ms	2.5 s	Static/morph	ha, an	Own	Zyg, Corr	29 (11 m, 18 f)
Dynamic expressions	Weyers et al., 2006	EMG	1 s	1.5 s; 500 ms time bins	Static/morph	ha, an, ne	Poser	Zyg, Corr	48 f
Familiarity	Fischer et al., 2012	FACS	n/a	n/a	Live setting	dis, pride	n/a	AU 4, 9, 10, 12, 43	112 f (56 dyads)
Familiarity	Häfner and Ijzerman, 2011 (Study 1)	EMG	2 s	2 s; 1 s time bins	Static (averaged)	ha, an	KDEF; Own	Zyg, Corr	23 dyads
Attitudes	Likowski et al., 2008	EMG	6 s	6 s	Static	ha, sad, ne	Poser	Zyg, Corr	25 f
Attitudes; empathy	Sims et al., 2012	EMG	4 s (apex at 2–3 s)	2000–4000 ms	Video	ha, an	TIGTE	Zyg, Corr	33 (7 m, 26 f)
Attitudes; empathy	Aguado et al., 2013	EMG	1 s	1 s; 100 ms time bins	Static	ha, an, ne	KDEF	Zyg, Corr	57 (9 m, 48 f)
Cooperation/competition empathy	Likowski et al., 2011a	EMG	6 s	6 s	Static	ha, an, sad, ne	Poser	Zyg, Corr, Orb	77 f
Cooperation/competition	Seibt et al., 2013	EMG	6 s	6 s	Static	ha, an, sad, ne	Poser	Zyg, Corr, Orb	84 f
Cooperation/competition	Weyers et al., 2009	EMG	8 s	8 s; 1 s time bins	Static	ha, sad, ne	Poser	Zyg, Corr	49 f
Fairness	Hofman et al., 2012	EMG	2/4 s	Bl 1+3: 4 s; Bl 2: 2 s	Morph + static	ha, an	EF2	Zyg, Corr	30 f
Power relationships	Carr et al., 2014	EMG	5 s	5 s; 500 ms time bins	Video	ha, an	MMI	Zyg, Corr	55 (82% f)
Ingroup vs. outgroup	McHugo et al., 1991	EMG	32–40 s	First 30 s	Video	ha, an, ne	Own	Zyg, Corr, Orb	100 (46 m, 54 f)
Ingroup vs. outgroup	McHugo et al., 1985	EMG	37–74 s	First (after 5 s) and last 15 s	Video	ha, an, fe, ne	Own	Zyg, Corr	40 (31 m, 9 f)
Ingroup vs. outgroup	Bourgeois and Hess, 2008 (Study 1)	EMG	*M* = 13.2 s	*M* = 13.2 s	Video	ha, an	Own	Zyg, Corr, Orb	54 (19 m, 25 f, 10?)
Ingroup vs. outgroup	Bourgeois and Hess, 2008 (Study 2)	EMG	Not specified	Not specified	Static	ha, an, sad	MSFDE	Zyg, Corr, Orb, Lev	60 m
Ingroup vs. outgroup	Mondillon et al., 2007 (Study 1)	RPA	400–1600 ms	n/a	Static	an, ne	MSFDE	n/a	47 f
Ingroup vs. outgroup	Mondillon et al., 2007 (Study 2)	RPA	400–1600 ms	n/a	Static	an, ne	MSFDE	n/a	41 f
Ingroup vs. outgroup	van der Schalk et al., 2011 (Study 1)	EMG	5 s	5 s	Static	ha, an, fe	Not specified	Corr, Orb	42 f
Ingroup vs. outgroup	van der Schalk et al., 2011 (Study 2)	FACS	~5 s (apex at 1 s)	~ 5 s	Video	ha, an, fe	Not specified	AU 4, 5, 6, 10, 12	153, (gender unspecified)
Social exclusion	(Kawamoto et al., 2014)	EMG, ERP	1 s	1 s	Static	ha, dis, ne	ATR	Zyg, Corr	42 (22 m, 20 f)

a The first keyword refers to the section where the study is mainly discussed, the second keyword to another mention within the review.

b EMG, Electromyogram; FACES, Facial Expression Coding System (Kring and Sloan, 2007); FACS, Facial Action Coding System (Ekman and Friesen, 1978a); RPA, response prediction accuracy; ERP, event related potential.

c per expression (where suitable time at apex of expression).

d total period of facial response measurement, if applicable, length of individual measurement bins. Ranges refer to time after stimulus onset (e.g., 300–1000 ms means measurement started 300 ms after stimulus onset and went on for 700 ms).

e morph: dynamic transition, usually from neutral to emotional expression, with first and last frame taken from photos or generated by the researcher, and transitional frames generated by the software.

f ha, happy; an, angry; ne, neutral; fe, fearful; dis, disgusted.

g Name of the stimulus set or software to generate the stimuli. Own, stimulus material created by the authors; MBFSS, MacBrain Face Stimulus Set (http://www.macbrain.org/resources.htm); JACFEE, Japanese and Caucasian Facial Expressions of Emotion (Matsumoto and Ekman, 1988); EF1, Unmasking the Face (Ekman and Friesen, 1978b); EF2, Pictures of Facial Affect (Ekman and Friesen, 1976); KDEF, Karolinska Directed Emotional Faces (Lundqvist et al., 1998); Poser, computer generated faces (Poser software, Curious Labs, Santa Cruz, CA); MSFDE, Montreal Set of Facial Displays of Emotion (Beaupré and Hess, 2005); TIGTE, Mindreading: The Interactive Guide to Emotions (Baron-Cohen et al., 2004); MMI, Facial Expression Database (Pantic et al., 2005); ATR, Facial Expression Database (DB99, ATR Promotions, Kyoto, Japan).

h Sites for EMG electrodes; Action units for FACS coding: Zyg, Zygomaticus major; Corr, Corrugator; Orb, Orbicularis Oculi; Dep, Depressor anguli oris, Lev, Levator labii; Front, Frontalis; KS07, Coding according to Kring and Sloan (2007); KJ79, response scoring according to Kraut and Johnston (1979).

#### Stimulus material

Differences across studies can, in part, be due to differences in the stimuli presented. For instance, fluency of processing is a source of positive affect, and can activate the Zygomaticus (Topolinski et al., 2009). Strong expressions, high visual contrast, familiarity and ingroup-status can increase fluency. Liking and attractiveness of faces also increase positive affect. The dynamic unfolding of expressions increases their salience and thereby guides attention. Furthermore, responses differ between, but also within expressions (e.g., polite vs. genuine smiles).

For these reasons, varied stimuli within the same study decrease statistical power (Westfall et al., 2014). To control for such variance, many studies used computer generated stimuli. Such stimuli can be introduced as avatars for concrete persons, allowing to investigate responses to known others in a highly controlled study. However, while responses to avatars and to photos are comparable (Spencer-Smith et al., 2001; Moser et al., 2007; Mühlberger et al., 2009), computer-generated stimuli, when falling in a specific “gap” in approaching realism (“Uncanny valley”), may engender negative reactions (de Borst and de Gelder, 2015).

In addition, human interaction entails different motivations and dynamics than passively looking at photos or avatars. For example, the presence of others enhances smiling expressions (Fridlund, 1991; Hess et al., 1995). But are reactions to stimuli on a screen and to interaction partners qualitatively the same, only more or less intense, or are they fundamentally different? Among the few articles using both kinds of situations, Heerey and Crossley (2013) found parallel effects of different types of smiles on the onset of mimicry reactions for FACS and EMG analyses of interaction and non-interaction situations, respectively. This is tentative evidence for qualitatively similar responses, but more direct evidence is needed. Thus, researchers should seek convergent evidence from well-controlled and from naturalistic settings.

#### Measurement and analysis

Having electrodes in the face is not very natural; thus, studies concerned with ecological validity employ FACS coding instead of EMG. This difference in methods, however, may also directly influence results. While only visible changes can be picked up by FACS, EMG can also pick up muscle activation that is invisible to the eye. The confound between natural situation and assessment method poses a problem: if EMG, but not FACS, reveals anger mimicry, it might be that in interactions, people do not show anger mimicry, or it might be that anger mimicry usually remains below the visibility level.

Many other methodological choices are likely to influence results of facial mimicry studies. Here, we highlight three. Facial mimicry studies vary considerably in the time period of measurement: some look at the time course of the activity changes, typically during the first second after stimulus onset, while others present the mean difference from baseline for a whole stimulus presentation period (e.g., 6 s). An important next step will be to study the time course of facial mimicry modulations in more detail. A second issue concerns the treatment of EMG data. This review examined whether social situations influence the mimicry of different emotions to different degrees. However, several studies tested angry against happy expressions, or standardized the difference scores before analysis, making it impossible to gauge the net-effect per emotion. The third issue concerns replicability of effects. Different method choices in different labs and sometimes considerable numbers of individual tests per study suggest that replication in this field is important and challenging.

### How do these processes develop?

A facial response to a facial expression, like any other response, can be based on a reflective process, or it can be impulsive, based on learned associations (Strack and Deutsch, 2004). Anger and happiness expressions are assumed to be unconditioned stimuli, and should therefore evoke unconditioned affective reactions (Tomkins, 1962; Ohman and Dimberg, 1978). In addition, other expressions are assumed to become conditioned stimuli, such as a competitor's sadness signaling victory (Lanzetta and Englis, 1989). Finally, operant conditioning can reinforce any response as long as it has positive consequences, which can explain a smiling reaction to one's partner's anger (Häfner and Ijzerman, 2011).

Unconditioned responses can be distinguished from learned responses on the basis of developmental studies with children of different age groups, which is a promising avenue for further research. To determine whether a response is given impulsively and thus the result of conditioning processes, or reflectively, researchers can vary the exposure time to the emotional expression (Sonnby-Borgström, 2002; Sonnby-Borgström et al., 2003), the response period observed (early vs. late reactions, see Moody et al., 2007), or concurrent cognitive load (Cannon et al., 2009). Further, combining EMG with fMRI measurement can help discover the pathways of facial mimicry (Likowski et al., 2012). Given the rapid progress in fMRI technology, this seems a particularly promising avenue for future research (Heller et al., 2014). If one assumes that there is a fast route to “mirror” facial expressions, the question at what stage of information processing this route is modulated becomes inevitable. Moody et al. (2007) propose that such effects can originate at perception, with heightened sensitivity to emotion-relevant expressions, at information processing, with amplification, biased interpretation and evaluation of relevant input or at response preparation, with pre-activation of relevant facial responses.

### The social encounter

As soon as individuals actually interact, they learn about each other (see Hofman et al., 2012; Aguado et al., 2013), which can, in turn, rectify pre-existing assumptions or change individuals' mood states. Factors such as the topic of the conversation, the facial mimicry of the sender, or the clarity, dynamic and type of facial signals will influence the emotional tone of the conversation and the cognitive and emotional states of the interaction partners. This, in turn, impacts the facial mimicry of the perceiver.

Furthermore, do interaction partners reciprocate the general amount of mimicry? Given that the brain's common currency is reward and punishment, it is likely that individuals distribute facial rewards and punishments just like other rewards and punishments in a tit-for-tat fashion (Axelrod and Hamilton, 1981), mimicking those who mimic them and stopping to mimic when the other is not mimicking them. Finally, relationship variables like power, attitudes, interdependence and fairness can be established, reinforced or mitigated through facial expressive and mimicry behavior. Studying facial mimicry in social encounters can help answer these questions. In addition, with refined software, it should be possible in the near future to test these propositions by manipulating the reciprocation of facial expressions via computer generated dynamic expressions shown by avatars or androids and triggered in real time by the participant's facial actions (see Bartlett and Whitehill, 2011; Littlewort et al., 2011; Hofree et al., 2014).

## Summary

Facial mimicry is embedded in the overall context. Congruent facial reactions are but one possible response to facial expressions. Another possibility is an incongruent response whose valence is opposite to that shown by the expresser. The selection of the reaction is determined by context-specific learning history. The collected evidence suggests that congruent facial expressions are by far not the reflex-like response they were once thought, and that facial reactions are not only quantitatively but also qualitatively modulated by contextual factors. To better understand facial mimicry, we have to study it in its social setting. Additionally, we have to design studies that shed light on functions and processes.

### Conflict of interest statement

The authors declare that the research was conducted in the absence of any commercial or financial relationships that could be construed as a potential conflict of interest.

## References

[B1] Achaibou A. Pourtois G. Schwartz S. Vuilleumier P. (2008). Simultaneous recording of EEG and facial muscle reactions during spontaneous emotional mimicry. Neuropsychologia 46, 1104–1113. 10.1016/j.neuropsychologia.2007.10.019 18068737

[B2] Adams R. B. Jr Kleck R. E. (2003). Perceived gaze direction and the processing of facial displays of emotion. Psychol. Sci. 14, 644–647. 10.1046/j.0956-7976.2003.psci_1479.x 14629700

[B3] Adams R. B. Jr Kleck R. E. (2005). Effects of direct and averted gaze on the perception of facially communicated emotion. Emotion 5, 3–11. 10.1037/1528-3542.5.1.3 15755215

[B4] Aguado L. Román F. J. Rodríguez S. Diéguez-Risco T. Romero-Ferreiro V. Fernández-Cahill M. (2013). Learning of facial responses to faces associated with positive or negative emotional expressions. Span. J. Psychol. 16, 1–10. 10.1017/sjp.2013.31 23866218

[B5] Ardizzi M. Sestito M. Martini F. Umiltà M. A. Ravera R. Gallese V. (2014). When age matters: differences in facial mimicry and autonomic responses to peers' emotions in teenagers and adults. PLoS ONE 9:e110763. 10.1371/journal.pone.0110763 25337916 PMC4206508

[B6] Axelrod R. Hamilton W. D. (1981). The evolution of cooperation. Science 211, 1390–1396. 10.1126/science.7466396 7466396

[B7] Bailenson J. N. Yee N. (2005). Digital chameleons: automatic assimilation of nonverbal gestures in immersive virtual environments. Psychol. Sci. 16, 814–819. 10.1111/j.1467-9280.2005.01619.x 16181445

[B8] Bailey P. E. Henry J. D. (2009). Subconscious facial expression mimicry is preserved in older adulthood. Psychol. Aging 24, 995–1000. 10.1037/a0015789 20025413

[B9] Bailey P. E. Henry J. D. Nangle M. R. (2009). Electromyographic evidence for age-related differences in the mimicry of anger. Psychol. Aging 24, 224–229. 10.1037/a0014112 19290755

[B10] Baron-Cohen S. Golan O. Wheelwright S. Hill J. (2004). Mindreading: The Interactive Guide to Emotions. London: Jessica Kingsley Publishers.

[B11] Baron-Cohen S. Wheelwright S. Hill J. Raste Y. Plumb I. (2001). The “Reading the mind in the eyes” Test revised version: a study with normal adults, and adults with Asperger syndrome or high-functioning autism. J. Child Psychol. Psychol. 42, 241–251. 10.1111/1469-7610.00715 11280420

[B12] Bartlett M. S. Whitehill J. (2011). Automated facial expression measurement: recent applications to basic research in human behavior, learning, and education, in The Oxford Handbook of Face Perception, eds Calder A. Rhodes G. Haxby J. V. Johnson M. H. (Oxford: Oxford University Press), 489–513.

[B13] Baumeister R. F. Leary M. R. (1995). The need to belong: desire for interpersonal attachments as fundamental human motivation. Psychol. Bull. 117, 497–529. 10.1037/0033-2909.117.3.497 7777651

[B14] Beaupré M. G. Hess U. (2005). Cross-cultural emotion recognition among Canadian ethnic groups. J. Cross Cult. Psychol. 36, 355–370. 10.1177/0022022104273656

[B15] Bölte S. (2005). Reading Mind in the Eyes Test für Erwachsene (dt. Fassung) von S. Baron-Cohen [Reading the Mind in the Eyes Test for Adults (German version) by S. Baron-Cohen]. Available online at: http://www.as-tt.de/assets/applets/Augentest_Erwachsene.pdf

[B16] Bourgeois P. Hess U. (2008). The impact of social context on mimicry. Biol. Psychol. 77, 343–352. 10.1016/j.biopsycho.2007.11.008 18164534

[B17] Bowlby J. (1969). Attachment and Loss, Vol. I: Attachment, Anxiety and Anger. London: Hogarth Press.

[B18] Brewer M. B. Gardner W. (1996). Who is this “We”? Levels of collective identity and self representations. J. Pers. Soc. Psychol. 71, 83–93. 10.1037/0022-3514.71.1.83

[B19] Cannon P. R. Hayes A. E. Tipper S. P. (2009). An electromyographic investigation of the impact of task relevance on facial mimicry. Cogn. Emot. 23, 918–929. 10.1080/02699930802234864

[B20] Cannon P. R. Schnall S. White M. (2011). Transgressions and expressions: affective facial muscle activity predicts moral judgments. Soc. Psychol. Pers. Sci. 2, 325–331. 10.1177/1948550610390525

[B21] Carr E. W. Winkielman P. Oveis C. (2014). Transforming the mirror: power fundamentally changes facial responding to emotional expressions. J. Exp. Psychol. 143, 997–1003. 10.1037/a0034972 24219023

[B22] Chartrand T. L. Bargh J. A. (1999). The chameleon effect: the perception–behavior link and social interaction. J. Pers. Soc. Psychol. 76, 893–910. 10.1037/0022-3514.76.6.893 10402679

[B23] Chartrand T. L. Maddux W. W. Lakin J. L. (2005). Beyond the perception-behavior link: the ubiquitous utility and motivational moderators of nonconscious mimicry, in The New Unconscious, eds Hassin R. R. Uleman J. S. Bargh J. A. (New York, NY: Oxford University Press), 334–361.

[B24] Chen M. Bargh J. A. (1999). Consequences of automatic evaluation: immediate behavioral predispositions to approach or avoid the stimulus. Pers. Soc. Psychol. Bull. 25, 215–224. 10.1177/0146167299025002007

[B25] Churchland P. S. Winkielman P. (2012). Modulating social behavior with oxytocin: how does it work? What does it mean? Horm. Behav. 61, 392–399. 10.1016/j.yhbeh.2011.12.003 22197271 PMC3312973

[B26] Clark M. S. Dubash P. Mills J. (1998). Interest in another's consideration of one's needs in communal and exchange relationships. J. Exp. Soc. Psychol. 34, 246–264. 10.1006/jesp.1998.13523746615

[B27] Darwin C. (1955 [1872]). The Expression of Emotions in Man Animals. New York, NY: Philosophical Library.

[B28] de Borst A. W. de Gelder B. (2015). Is it the real deal? Perception of virtual characters versus humans: an affective cognitive neuroscience perspective. Front. Psychol. 6:576. 10.3389/fpsyg.2015.00576 26029133 PMC4428060

[B29] de Waal F. B. (2008). Putting the altruism back into altruism: the evolution of empathy. Annu. Rev. Psychol. 59, 279–300. 10.1146/annurev.psych.59.103006.093625 17550343

[B30] Decety J. Jackson P. L. (2004). The functional architecture of human empathy. Behav. Cogn. Neurosci. Rev. 3, 406–412. 10.1177/1534582304267187 15537986

[B31] Dickens L. DeSteno D. (2014). Pride attenuates nonconscious mimicry. Emotion 14, 7–11. 10.1037/a0035291 24491248

[B32] Dimberg U. (1997). Social fear and expressive reactions to social stimuli. Scand. J. Psychol. 38, 171–174. 10.1111/1467-9450.0002411408051

[B33] Dimberg U. Christmanson L. (1991). Facial reactions to facial expressions in subjects high and low in public speaking fear. Scand. J. Psychol. 32, 246–253. 10.1111/j.1467-9450.1991.tb00875.x 1759142

[B34] Dimberg U. Lundquist L. O. (1990). Gender differences in facial reactions to facial expressions. Biol. Psychol. 30, 151–159. 10.1016/0301-0511(90)90024-Q 2285765

[B35] Dimberg U. Thunberg M. (1998). Rapid facial reactions to emotional facial expressions. Scand. J. Psychol. 39, 39–45. 10.1111/1467-9450.00054 9619131

[B36] Dimberg U. Thunberg M. (2007). Speech anxiety and rapid emotional reactions to angry and happy facial expressions. Scand. J. Psychol. 48, 321–328. 10.1111/j.1467-9450.2007.00586.x 17669222

[B37] Dimberg U. Thunberg M. Elmehed K. (2000). Unconscious facial reactions to emotional facial expressions. Psychol. Sci. 11, 86–89. 10.1111/1467-9280.00221 11228851

[B38] Dimberg U. Thunberg M. Grunedal S. (2002). Facial reactions to emotional stimuli: automatically controlled emotional responses. Cogn. Emot. 16, 449–472. 10.1080/0269993014300035615652310

[B39] Dimberg U. Andréasson P. Thunberg M. (2011). Emotional empathy and facial reactions to facial expressions. J. Psychophysiol. 25, 26–31. 10.1027/0269-8803/a000029

[B40] Eisenegger C. Haushofer J. Fehr E. (2011). The role of testosterone in social interaction. Trends Cogn. Sci. 15, 263–271. 10.1016/j.tics.2011.04.008 21616702

[B41] Ekman P. Friesen W. V. (1976). Pictures of Facial Affect. Palo Alto, CA: Consulting Psychologists Press.

[B42] Ekman P. Friesen W. V. (1978a). The Facial Action Coding System. PaloAlto, CA: Consulting Psychologists Press.

[B43] Ekman P. Friesen W. V. (1978b). Unmasking the Face. Englewood Cliffs, NJ: Prentice Hall.

[B44] Field T. M. Woodson R. Greenberg R. Cohen D. (1982). Discrimination and imitation of facial expressions by neonates. Science 218, 179–181. 10.1126/science.7123230 7123230

[B45] Fiske A. P. (2004). Four modes of constituting relationships: consubstantial assimilation; space, magnitude, time and force; concrete procedures; abstract symbolism, in Relational Models Theory: A Contemporary Overview, ed Haslam N. (Mahwah, NJ: Lawrence Erlbaum Associates), 61–146.

[B46] Fiske A. P. Fiske S. T. (2007). Social relations in our species and our cultures, in Handbook of Cultural Psychology, eds Kitayama S. Cohen D. (New York, NY: The Guilford Press), 283–306.

[B47] Fischer A. H. Becker D. Veenstra L. (2012). Emotional mimicry in social context: the case of disgust and pride. Front. Psychol. 3:475. 10.3389/fpsyg.2012.00475 23130013 PMC3487425

[B48] Fraley R. C. Shaver P. R. (1997). Adult attachment and the suppression of unwanted thoughts. J. Pers. Soc. Psychol. 73, 1080–1091. 10.1037/0022-3514.73.5.1080 9364762

[B49] Fridlund A. J. (1991). Sociality of solitary smiling: potentiation by an implicit audience. J. Pers. Soc. Psychol. 60, 229–240. 10.1037/0022-3514.60.2.229

[B50] Fridlund A. J. Cacioppo J. T. (1986). Guidelines for human electromyographic research. Psychophysiology 23, 567–589. 10.1111/j.1469-8986.1986.tb00676.x 3809364

[B51] Goldman A.I. Sripada C. S. (2005). Simulationist models of face-based emotion recognition. Cognition 94, 193–213. 10.1016/j.cognition.2004.01.005 15617671

[B52] Graham R. Labar K. S. (2012). Neurocognitive mechanisms of gaze-expression interactions in face processing and social attention. Neuropsychologia 50, 553–566. 10.1016/j.neuropsychologia.2012.01.019 22285906 PMC3309061

[B53] Green J. D. Sedikides C. (1999). Affect and self-focused attention revisited: the role of affect orientation. Pers. Soc. Psychol. Bull. 25, 104–119. 10.1177/0146167299025001009

[B54] Gregg A. P. Seibt B. Banaji M. R. (2006). Easier done than undone: asymmetry in the malleability of implicit preferences. J. Pers. Soc. Psychol. 90, 1–20. 10.1037/0022-3514.90.1.1 16448307

[B55] Gross J. J. (2014). Emotion regulation: conceptual and empirical foundations, in Handbook of Emotion Regulation, 2nd Edn., ed Gross J. J. (Guilford; New York: The Guilford Press), 3–20.

[B56] Häfner M. Ijzerman H. (2011). The face of love: spontaneous accommodation as social emotion regulation. Pers. Soc. Psychol. Bull. 37, 1551–1563. 10.1177/0146167211415629 21778468

[B57] Harrison N. A. Morgan R. Critchley H. D. (2010). From facial mimicry to emotional empathy: a role for norepinephrine? Soc. Neurosci. 5, 393–400. 10.1080/17470911003656330 20486012 PMC2913325

[B58] Heerey E. A. Crossley H. M. (2013). Predictive and reactive mechanisms in smile reciprocity. Psychol. Sci. 24, 1446–1455. 10.1177/0956797612472203 23744875

[B59] Heller A. S. Greischar L. L. Honor A. Anderle M. J. Davidson R. J. (2014). Simultaneous acquisition of corrugator electromyography and functional magnetic resonance imaging: a new method for objectively measuring affect and neural activity concurrently. Neuroimage 58, 930–934. 10.1016/j.neuroimage.2011.06.057 21742043 PMC3206735

[B60] Hermans E. J. Putman P. van Honk J. (2006). Testosterone administration reduces empathetic behavior: a facial mimicry study. Psychoneuroendocrinology 31, 859–866. 10.1016/j.psyneuen.2006.04.002 16769178

[B61] Hermans E. J. van Wingen G. Bos P. A. Putman P. van Honk J. (2009). Reduced spontaneous facial mimicry in women with autistic traits. Biol. Psychol. 80, 348–353. 10.1016/j.biopsycho.2008.12.002 19136041

[B62] Hess U. (2001). The communication of emotion, in Emotion, Qualia and Consciousness, ed Kazniak A. (Singapore: World Scientific Publishing), 386–396.

[B63] Hess U. Banse R. Kappas A. (1995). The intensity of facial expression is determined by underlying affective state and social situation. J. Pers. Soc. Psychol. 69, 280–288. 10.1037/0022-3514.69.2.280

[B64] Hess U. Bourgeois P. (2010). You smile - I smile: emotion expression in social interaction. Biol. Psychol. 84, 514–520. 10.1016/j.biopsycho.2009.11.001 19913071

[B65] Hess U. Fischer A. (2013). Emotional mimicry as social regulation. Pers. Soc. Psychol. Rev. 17, 142–157. 10.1177/1088868312472607 23348982

[B66] Hess U. Fischer A. (2014). Emotional mimicry: why and when we mimic emotions. Soc. Pers. Psychol. Compass 8, 45–57. 10.1111/spc3.12083

[B67] Hess U. Philippot P. Blairy S. (1998). Facial reactions to emotional facial expressions: affect or cognition? Cogn. Emot. 12, 509–532. 10.1080/026999398379547

[B68] Hinsz V. B. Tomhave J. A. (1991). Smile and (half) the world smiles with you, frown and you frown alone. Pers. Soc. Psychol. Bull. 17, 586–592. 10.1177/0146167291175014

[B69] Hofman D. Bos P. A. Schutter D. J. van Honk J. (2012). Fairness modulates non-conscious facial mimicry in women. Proc. R. Soc. B Biol. Sci. 279, 3535–3539. 10.1098/rspb.2012.0694 22648158 PMC3396901

[B70] Hofree G. Ruvolo P. Bartlett M. S. Winkielman P. (2014). Bridging the mechanical and the human mind: spontaneous mimicry of a physically present android. PLoS ONE 9:e99934. 10.1371/journal.pone.0099934 25036365 PMC4103778

[B71] Hühnel I. Fölster M. Werheid K. Hess U. (2014). Empathic reactions of younger and older adults: no age related decline in affective responding. J. Exp. Soc. Psychol. 50, 36–143. 10.1371/journal.pone.0099934

[B72] Jack R. E. Garrod O. G. Yu H. Caldara R. Schyns P. G. (2012). Facial expressions of emotion are not culturally universal. Proc. Natl. Acad. Sci. U.S.A. 109, 7241–7244. 10.1073/pnas.1200155109 22509011 PMC3358835

[B73] Kanat M. Heinrichs M. Domes G. (2014). Oxytocin and the social brain: neural mechanisms and perspectives in human research. Brain Res. 1580, 160–171. 10.1016/j.brainres.2013.11.003 24216134

[B74] Kawamoto T. Nittono H. Ura M. (2014). Social exclusion induces early-stage perceptual and behavioral changes in response to social cues. Soc. Neurosci. 9, 174–185. 10.1080/17470919.2014.883325 24499456

[B75] Korb S. With S. Niedenthal P. Kaiser S. Grandjean D. (2014). The perception and mimicry of facial movements predict judgments of smile authenticity. PLoS ONE 9:e99194. 10.1371/journal.pone.0099194 24918939 PMC4053432

[B76] Korb S. Grandjean D. Scherer K. (2010). Timing and voluntary suppression of facial mimicry to smiling faces in a Go/NoGo task—An EMG study. Biol. Psychol. 85, 347–349. 10.1016/j.biopsycho.2010.07.012 20673787

[B77] Koriat A. Nussinson R. (2009). Attributing study effort to data-driven and goal-driven effects: implications for metacognitive judgments. J. Exp. Psychol 35, 1338–1343. 10.1037/a0016374 19686026

[B78] Kraut R. E. Johnston R. E. (1979). Social and emotional messages of smiling: an ethological approach. J. Pers. Soc. Psychol. 37, 1539–1553. 10.1037/0022-3514.37.9.1539

[B79] Krieglmeyer R. Deutsch R. (2013). Approach does not equal approach: angry facial expressions evoke approach only when it serves aggression. Soc. Psychol. Pers. Sci. 4, 607–614. 10.1177/1948550612471060

[B80] Kring A. M. Sloan D. M. (2007). The Facial Expression Coding System (FACES): development, validation, and utility. Psychol. Assess. 19, 210–224. 10.1037/1040-3590.19.2.210 17563202

[B81] Lakin J. L. Chartrand T. L. (2003). Using nonconscious behavioral mimicry to create affiliation and rapport. Psychol. Sci. 14, 334–339. 10.1111/1467-9280.14481 12807406

[B82] Lakin J. L. Jefferis V. E. Cheng C. M. Chartrand T. L. (2003). The chameleon effect as social glue: evidence for the evolutionary significance of nonconscious mimicry. J. Nonverbal Behav. 27, 145–162. 10.1023/A:1025389814290

[B83] Lang P. J. Greenwald M. Bradley M. M. Hamm A. O. (1993). Looking at pictures: evaluative, facial, visceral, and behavioral responses. Psychophysiology 30, 261–273. 10.1111/j.1469-8986.1993.tb03352.x 8497555

[B84] Lanzetta J. T. Englis B. G. (1989). Expectations of cooperation and competition and their effects on observers' vicarious emotional responses. J. Pers. Soc. Psychol. 56, 543–554. 10.1037/0022-3514.56.4.543

[B85] Larsen J. T. Norris C. J. Cacioppo J. T. (2003). Effects of positive and negative affect on electromyographic activity over zygomaticus major and corrugator supercilii. Psychophysiology 40, 776–785. 10.1111/1469-8986.00078 14696731

[B86] Likowski K. U. Mühlberger A. Gerdes A. B. M. Wieser M. J. Pauli P. Weyers P. (2012). Facial mimicry and the mirror neuron system: simultaneous acquisition of facial electromyography and functional magnetic resonance imaging. Front. Hum. Neurosci. 6:214. 10.3389/fnhum.2012.00214 22855675 PMC3405279

[B87] Likowski K. U. Mühlberger A. Seibt B. Pauli P. Weyers P. (2008). Modulation of facial mimicry by attitudes. J. Exp. Soc. Psychol. 44, 1065–1072. 10.1016/j.jesp.2007.10.007

[B88] Likowski K. U. Mühlberger A. Seibt B. Pauli P. Weyers P. (2011a). Processes underlying congruent and incongruent facial reactions to emotional facial expressions. Emotion 11, 457–467. 10.1037/a0023162 21668100

[B89] Likowski K. U. Weyers P. Seibt B. Stöhr C. Pauli P. Mühlberger A. (2011b). Sad and lonely? Sad mood suppresses facial mimicry. J. Nonverbal Behav. 35, 101–117. 10.1007/s10919-011-0107-4

[B90] Littlewort G. Whitehill J. Wu T. Fasel I. Frank M. Movellan J. Bartlett M. (2011). The computer expression recognition toolbox, in 2011 IEEE International Conference on Automatic Face and Gesture Recognition (FG2011) (Santa Barbara, CA), 334–361. 10.1109/FG.2011.5771214

[B91] Luu P. Tucker D. M. Derryberry D. (1998). Anxiety and the motivational basis of working memory. Cogn. Ther. Res. 22, 577–594. 10.1023/A:101874212025517675916

[B92] Lundqvist D. Flykt A. Öhman A. (1998). The Karolinska Directed Emotional Faces. Stockholm: Karolinska Institute, Psychology section, Department of Clinical Neuroscience.

[B93] Mackie D. M. Devos T. Smith E. R. (2000). Intergroup emotions: explaining offensive action tendencies in an intergroup context. J. Pers. Soc. Psychol. 79, 602–616. 10.1037/0022-3514.79.4.602 11045741

[B94] Maringer M. Krumhuber E. G. Fischer A. H. Niedenthal P. M. (2011). Beyond smile dynamics: mimicry and beliefs in judgments of smiles. Emotion 11, 181–187. 10.1037/a0022596 21401238

[B95] Matsumoto D. Ekman P. (1988). The Japanese and Caucasian Facial Expressions of Emotion (JACFEE) and Neutrals (JACNeuF) (Slides). San Francisco, CA: Intercultural and Emotion Research Laboratory, Department of Psychology, San Francisco State University.

[B96] McHugo G. J. Lanzetta J. T. Bush L. K. (1991). The effect of attitudes on emotional reactions to expressive displays of political leaders. J. Nonverbal Behav. 15, 19–41. 10.1007/BF00997765

[B97] McHugo G. J. Lanzetta J. T. Sullivan D. G. Masters R. D. Englis B. G. (1985). Emotional reactions to a political leader's expressive displays. J. Pers. Soc. Psychol. 49, 1513–1529. 10.1037/0022-3514.49.6.1513

[B98] Mehrabian A. Epstein N. (1972). A measure of emotional empathy. J. Pers. 40, 525–543. 10.1111/j.1467-6494.1972.tb00078.x 4642390

[B99] Meltzoff A. N. Moore M. K. (1989). Imitation in newborn infants: exploring the range of gestures imitated and the underlying mechanisms. Dev. Psychol. 25, 954–962. 10.1037/0012-1649.25.6.954 25147405 PMC4137867

[B100] Messinger D. S. Fogel A. Dickson K. (2001). All smiles are positive, but some smiles are more positive than others. Dev. Psychol. 37, 642–653. 10.1037/0012-1649.37.5.642 11552760

[B101] Mondillon L. Niedenthal P. M. Gil S. Droit-Volet S. (2007). Imitation of in-group versus out-group members' facial expressions of anger: a test with a time perception task. Soc. Neurosci. 2, 223–237. 10.1080/17470910701376894 18633817

[B102] Moody E. J. McIntosh D. N. Mann L. J. Weisser K. R. (2007). More than mere mimicry? The influence of emotion on rapid facial reactions to faces. Emotion 7, 447–457. 10.1037/1528-3542.7.2.447 17516821

[B103] Moser E. Derntl B. Robinson S. Fink B. Gur R. C. Grammer K. (2007). Amygdala activation at 3T in response to human and avatar facial expressions of emotions. J. Neurosci. Meth. 161, 126–133. 10.1016/j.jneumeth.2006.10.016 17126910

[B104] Mühlberger A. Wieser M. J. Herrmann M. J. Weyers P. Tröger C. Pauli P. (2009). Early cortical processing of natural and artificial emotional faces differs between lower and higher social anxious persons. J. Neural Transm. 116, 735–746. 10.1007/s00702-008-0108-6 18784899

[B105] Mühlberger A. Wieser M. J. Gerdes A. B. Frey M. C. Weyers P. Pauli P. (2011). Stop looking angry and smile, please: start and stop of the very same facial expression differentially activate threat- and reward-related brain networks. Soc. Cogn. Affect. Neurosci. 6, 321–329. 10.1093/scan/nsq039 20460301 PMC3110429

[B106] Navarrete C. D. MacDonald M. M. Molina L. E. Sidanius J. (2010). Prejudice at the nexus of race and gender: an outgroup male target hypothesis. J. Pers. Soc. Psychol. 98, 933–945. 10.1037/a0017931 20515248

[B107] Neal D. T. Chartrand T. L. (2011). Embodied emotion perception amplifying and dampening facial feedback modulates emotion perception accuracy. Soc. Psychol. Pers. Sci. 2, 673–678. 10.1177/1948550611406138

[B108] Neumann R. Strack F. (2000). Approach and avoidance: the influence of proprioceptive and exteroceptive cues on encoding of affective information. J. Pers. Soc. Psychol. 79, 39–48. 10.1037/0022-3514.79.1.39 10909876

[B109] Neumann R. Hülsenbeck K. Seibt B. (2004). Attitudes towards people with AIDS and avoidance behavior: automatic and reflective bases of behavior. J. Exp. Soc. Psychol. 40, 543–550. 10.1016/j.jesp.2003.10.006

[B110] Niedenthal P. M. Mermillod M. Maringer M. Hess U. (2010). The Simulation of Smiles (SIMS) model: embodied simulation and the meaning of facial expression. Behav. Brain Sci. 33, 417–433. 10.1017/S0140525X10000865 21211115

[B111] Oberman L. M. Winkielman P. Ramachandran V. S. (2007). Face to face: blocking facial mimicry can selectively impair recognition of emotional expressions. Soc. Neurosci. 2, 167–178. 10.1080/17470910701391943 18633815

[B112] Ohman A. Dimberg U. (1978). Facial expressions as conditioned stimuli for electrodermal responses: a case of “preparedness”? J. Pers. Soc. Psychol. 36, 1251–1258. 10.1037/0022-3514.36.11.1251 745035

[B113] Pantic M. Valstar M. F. Rademaker R. Maat L. (2005). Web-based database for facial expression analysis, in Proceedings of the IEEE International Conference on Multmedia and Expo (ICME'05) (Amsterdam).

[B114] Paul G. L. (1966). Insight Versus Desensitization in Psychotherapy. Stanford: Stanford University Press.

[B115] Prinz W. (1990). A common coding approach to perception and action, in Relationships between Perception and Action, eds Neumann O. Prinz W. (Berlin: Springer), 167–201.

[B116] Rychlowska M. Miyamoto Y. Matsumoto D. Hess U. Gilboa-Schechtman E. Kamble S.. (2015). Heterogeneity of long-history migration explains cultural differences in reports of emotional expressivity and the functions of smiles. Proc. Natl. Acad. Sci. U.S.A. 112, E2429–E2436. 10.1073/pnas.1413661112 25902500 PMC4434713

[B117] Rychlowska M. Zinner L. Musca S. C. Niedenthal P. (2012). From the eye to the heart: eye contact triggers emotion simulation, in Proceedings of the 4th Workshop on Eye Gaze in Intelligent Human Machine Interaction (New York, NY: ACM Digital Library), 5 10.1145/2401836.2401841

[B118] Rychlowska M. Canadas E. Wood A. Krumhuber E. Fischer A. Niedenthal P. (2014). Blocking mimicry makes true and false smiles look the same. PLoS ONE 9:e90876. 10.1371/journal.pone.0090876 24670316 PMC3966726

[B119] Rymarczyk K. Biele C. Grabowska A. Majczynski H. (2011). EMG activity in response to static and dynamic facial expressions. Int. J. Psychophysiol. 79, 330–333. 10.1016/j.ijpsycho.2010.11.001 21074582

[B120] Sanna L. J. Schwarz N. Small E. M. (2002). Accessibility experiences and the hindsight bias: I knew it all along versus it could never have happened. Mem. Cogn. 30, 1288–1296. 10.3758/BF03213410 12661859

[B121] Sato W. Fujimura T. Suzuki N. (2008). Enhanced facial EMG to dynamic facial expressions. Int. J. Psychophysiol. 70, 70–74. 10.1016/j.ijpsycho.2008.06.001 18598725

[B122] Sato W. Yoshikawa S. (2007). Spontaneous facial mimicry in response to dynamic facial expressions. Cognition 104, 1–18. 10.1016/j.cognition.2006.05.001 16780824

[B123] Schrammel F. Pannasch S. Graupner S.-T. Mojzisch A. Velichowsky B. M. (2009). Virtual friend or threat? The effects of facial expression and gaze interaction on psychophysiological responses and emotional experience. Psychophysiology 46, 922–931. 10.1111/j.1469-8986.2009.00831.x 19470127

[B124] Schubert T. W. Häfner M. (2003). Contrast from social stereotypes in automatic behavior. J. Exp. Soc. Psychol. 39, 577–584. 10.1016/S0022-1031(03)00034-9

[B125] Schwarz N. Clore G. (1996). Feelings and phenomenal experiences. in Social Psychology: Handbook of Basic Principles, eds Higgins E. T. Kruglanski A. W. (New York, NY: The Guilford Press), 433–465.

[B126] Sedikides C. (1992). Mood as determinant of attentional focus. Cogn. Emot. 6, 129–148. 10.1080/02699939208411063

[B127] Seibt B. Weyers P. Likowski K. U. Pauli P. Mühlberger A. Hess U. (2013). Non-conscious interdependence priming modulates congruent and incongruent facial reactions to emotional displays. Soc. Cogn. 31, 613–631. 10.1521/soco.2013.31.5.613

[B128] Seibt B. Neumann R. Nussinson R. Strack F. (2008). Movement direction or change in distance? Self- and object-related approach–avoidance motions. J. Exp. Soc. Psychol. 44, 713–720. 10.1016/j.jesp.2007.04.013

[B129] Shahrestani S. Kemp A. H. Guastella A. J. (2013). The impact of a single administration of intranasal oxytocin on the recognition of basic emotions in humans: a meta-analysis. Neuropsychopharmacology 38, 1929–1936. 10.1038/npp.2013.86 23575742 PMC3746698

[B130] Shamay-Tsoory S. G. Aharon-Peretz J. Perry D. (2009). Two systems for empathy: a double dissociation between emotional and cognitive empathy in inferior frontal gyrus versus ventromedial prefrontal lesions. Brain 132, 617–627. 10.1093/brain/awn279 18971202

[B131] Sims T. B. van Reekum C. M. Johnstone T. Chakrabarti B. (2012). How reward modulates mimicry: EMG evidence of greater facial mimicry of more rewarding happy faces. Psychophysiology 49, 998–1004. 10.1111/j.1469-8986.2012.01377.x 22563935

[B132] Smith E. R. Seger C. R. Mackie D. M. (2007). Can emotions be truly group level? Evidence regarding four conceptual criteria. J. Pers. Soc. Psychol. 93, 431–446. 10.1037/0022-3514.93.3.431 17723058

[B133] Sonnby-Borgström M. (2002). Automatic mimicry reactions as related to differences in emotional empathy. Scand. J. Psychol. 43, 433–443. 10.1111/1467-9450.00312 12500783

[B134] Sonnby-Borgström M. Jönsson P. (2004). Dismissing-avoidant pattern of attachment and mimicry reactions at different levels of information processing. Scand. J. Psychol. 45, 103–113. 10.1111/j.1467-9450.2004.00385.x 15016264

[B135] Sonnby-Borgström M. Jönsson P. Svensson O. (2003). Emotional empathy as related to mimicry reactions at different levels of information processing. J. Nonverbal Behav. 27, 3–23. 10.1023/A:1023608506243

[B136] Soussignan R. Chadwick M. Philip L. Conty L. Dezecache G. Grèzes J. (2013). Self-relevance appraisal of gaze direction and dynamic facial expressions: effects on facial electromyographic and autonomic reactions. Emotion 13, 330–337. 10.1037/a0029892 22985342

[B137] Spencer-Smith J. Wild H. Innes-Ker A. H. Townsend J. Duffy C. Edwards C.. (2001). Making faces: creating three-dimensional parameterized models of facial expression. Behav. Res. Methods Instr. Comput. 33, 115–123. 10.3758/BF03195356 11447663

[B138] Stel M. van Knippenberg A. (2008). The role of facial mimicry in the recognition of affect. Psychol. Sci. 19, 984–985. 10.1111/j.1467-9280.2008.02188.x 19000207

[B139] Stepper S. Strack F. (1993). Proprioceptive determinants of affective and nonaffective feelings. J. Pers. Soc. Psychol. 64, 211–220. 10.1037/0022-3514.64.2.211

[B140] Strack F. Deutsch R. (2004). Reflective and impulsive determinants of social behavior. Pers. Soc. Psychol. Rev. 8, 220–247. 10.1207/s15327957pspr0803_1 15454347

[B141] Strack F. Neumann R. (2000). Furrowing the brow may undermine perceived fame: the role of facial feedback in judgments of celebrity. Pers. Soc. Psychol. B 26, 762–768. 10.1177/0146167200269002

[B142] Tiedens L. Z. Fragale A. R. (2003). Power moves: complementarity in dominant and submissive nonverbal behavior. J. Pers. Soc. Psychol. 84, 558–568. 10.1037/0022-3514.84.3.558 12635916

[B143] Tomkins S. S. (1962). Affect, Imagery, Consciousness: Vol. I. The Positive Affects. New York, NY: Springer.

[B144] Topolinski S. Likowski K. U. Weyers P. Strack F. (2009). The face of fluency: semantic coherence automatically elicits a specific pattern of facial muscle reactions. Cogn. Emot. 23, 260–271. 10.1080/02699930801994112

[B145] Topolinski S. Strack F. (2015). Corrugator activity confirms immediate negative affect in surprise. Front. Psychol. 6:134. 10.3389/fpsyg.2015.00134 25762956 PMC4329793

[B146] van der Schalk J. Fischer A. H. Doosje B. J. Wigboldus D. Hawk S. T. Hess U.. (2011). Congruent and incongruent responses to emotional displays of ingroup and outgroup. Emotion 11, 286–298. 10.1037/a0022582 21500898

[B147] van Honk J. Tuiten A. Hermans E. Putman P. Koppeschaar H. Thijssen J.. (2001). A single administration of testosterone induces cardiac accelerative responses to angry faces in healthy young women. Behav. Neurosci. 115, 238–242. 10.1037/0735-7044.115.1.238 11256447

[B148] van Honk J. Schutter D. J. L. G. (2007). Testosterone reduces conscious detection of signals serving social correction. Implicat. Antisocial Behav. Psychol. Sci. 18, 663–667. 10.1111/j.1467-9280.2007.01955.x 17680933

[B149] van Vugt M. De Cremer D. Janssen D. P. (2007). Gender differences in cooperation and competition: the male-warrior hypothesis. Psychol. Sci. 18, 19–23. 10.1111/j.1467-9280.2007.01842.x 17362372

[B150] Vanman E. J. Saltz J. L. Nathan L. R. Warren J. A. (2004). Racial discrimination by low-prejudiced Whites. Facial movements as implicit measures of attitudes related to behavior. Psychol. Sci. 15, 711–714. 10.1111/j.0956-7976.2004.00746.x 15482441

[B151] Vrana S. R. Gross D. (2004). Reactions to facial expressions: effects of social context and speech anxiety on responses to neutral, anger, and joy expressions. Biol. Psychol. 66, 63–78. 10.1016/j.biopsycho.2003.07.004 15019171

[B152] Westfall J. Kenny D. A. Judd C. M. (2014). Statistical power and optimal design in experiments in which samples of participants respond to samples of stimuli. J. Exp. Psychol. 143, 2020–2045. 10.1037/xge0000014 25111580

[B153] Weyers P. Mühlberger A. Hefele C. Pauli P. (2006). Electromyographic responses to static and dynamic avatar emotional facial expressions. Psychophysiology 43, 450–453. 10.1111/j.1469-8986.2006.00451.x 16965606

[B154] Weyers P. Mühlberger A. Kund A. Hess U. Pauli P. (2009). Modulation of facial reactions to avatar emotional faces by nonconscious competition priming. Psychophysiology 46, 328–335. 10.1111/j.1469-8986.2008.00771.x 19207205

[B155] Wood J. V. Saltzberg J. A. Goldsamt L. A. (1990). Does affect induce self-focused attention? J. Pers. Soc. Psychol. 58, 899–908. 10.1037/0022-3514.58.5.899 2348375

[B156] Yuki M. Maddux W. W. Masuda T. (2007). Are the windows to the soul the same in the East and West? Cultural differences in using the eyes and mouth as cues to recognize emotions in Japan and the United States. J. Exp. Soc. Psychol. 43, 303–311. 10.1016/j.jesp.2006.02.004

